# Recent Advances in Organic Phototransistors: Nonvolatile Memory, Artificial Synapses, and Photodetectors

**DOI:** 10.1002/smsc.202100109

**Published:** 2022-01-27

**Authors:** Yan-Cheng Lin, Wei-Chen Yang, Yun-Chi Chiang, Wen-Chang Chen

**Affiliations:** ^1^ Department of Chemical Engineering National Taiwan University Taipei 10617 Taiwan; ^2^ Advanced Research Center of Green Materials Science and Technology National Taiwan University Taipei 10617 Taiwan

**Keywords:** field-effect transistors, floating gate electrets, photomemory, sensors, synaptic transistors

## Abstract

Recent research interest in organic field‐effect transistor (FET) memory has shifted to the functionality of photoprogramming in terms of its potential uses in multibit data storage and light‐assisted encryption and its low‐energy consumption and broad response to various optical bands. Phototransistor memory can be modulated through both electrical stress and light illumination, allowing it to function as an orthogonal operation method without mutual interference. Herein, the basic design concepts, requirements, and architectures of phototransistor memory are introduced. Design architectures such as channel‐only, channel‐with‐photogate, photochromatic channel devices and floating gate, photoactive polymer, and organic molecule‐based electrets are systematically categorized. The operational mechanism and impact of effective combinations of channels and electrets are reviewed to provide a fundamental understanding of photoprogramming as well as its potential future developmental applications as nonvolatile memory. Furthermore, recent advances in phototransistors and their diverse applications, including nonvolatile memory, artificial synapses, and photodetectors, are summarized. Finally, the outlook for the future development of phototransistors is briefly discussed. A comprehensive picture of the recent progress in phototransistors is provided.

## Graphical abstract

Herein, the basic design concepts and architectures of phototransistors are introduced. Design strategies involving channel‐only and channel‐with‐photogate devices as well as devices using floating gate, photoactive polymer, and organic molecule electrets are systematically categorized. Recent advances in phototransistors and their diverse applications: nonvolatile memory, artificial synapses, and photodetectors, are summarized. This review sheds light on the future development of phototransistors.

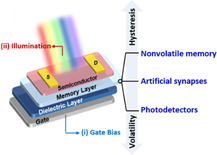

## 1 Introduction

In the past decade, nonvolatile memory devices have emerged as the most innovative products of the electronics community. With regard to nonvolatile memory devices, they can be majorly categorized into two or three‐terminal devices. In terms of two‐terminal devices, there are memristors, ferroelectric memristors, phase‐change, or diode‐type devices, that their conductance can be modified incrementally by controlling charge or ion flux through them.^[^ ^1^, ^2^, ^3^ ^]^ Two‐terminal devices represent the merits of simple structure and compatibility with high‐density crossbar array architecture. However, their signal transmission and programming functions cannot be performed concomitantly, and this demerit further confines its application in artificial synapse owing to the mutually interfered learning and forgetting functions. In contrast to two‐terminal devices, three‐terminal devices can receive and read stimuli concurrently, and they can be divided into memristor and transistor‐type devices.^[^ ^4^, ^5^, ^6^ ^]^ Memristor is fabricated by connecting two memristors to the same bottom electrode and the memristor functions are achieved by switching the memristors in parallel.^[^ ^4^ ^]^ Transistors show highly concentrated carrier concentrations in channels, which is conducive to higher sensitivity than memristors. Notably, transistors are capable of producing spatiotemporal effects by installing multiple gates to obtain signals from different sources. Therefore, three‐terminal devices, in terms of transistors, are more competent in versatile optoelectronic applications, including nonvolatile memory, artificial synapses, and detectors.^[^ ^7^, ^8^ ^]^ With regard to the material systems of transistors, inorganic materials such as perovskite quantum dots (QDs) or graphene, transition metal dichalcogenides with intralayer covalent bonding, and interlayer van der Waals interaction have the advantages of good stability, high carrier mobility, and moderate bandgap,^[^ ^9^, ^10^, ^11^, ^12^, ^13^, ^14^, ^15^ ^]^ whereas organic materials have the merits of having large free volumes to mediate migration of abundant ions and easy tunability by modification of chemical structures and physical properties such as crystallinity and compatibility with solution processing that enables easy and scalable fabrication.^[^ ^16^, ^17^, ^18^, ^19^, ^20^, ^21^ ^]^ These merits overcome the obstacles of inorganic materials requiring complicated manufacturing technologies and expensive facilities, and they are also very frangible. Notably, organic field‐effect transistor (OFET) memory has attracted extensive attention owing to its advantages in terms of nondestructive readout, easily integrated structures, good compatibility with complementary metal oxide semiconductors (CMOS), light weight, and flexibility for use in next‐generation wearable devices.^[^ ^22^, ^23^, ^24^ ^]^ The rapid development of the Internet of Things (IoT) and artificial intelligence and the urgent demand for information storage (or big data) have significantly accelerated the growth of computational calculation. However, there remains a significant gap between the amount of data generated and the capacities of storage media that constitutes a so‐called “memory wall” bottleneck in the development of digital technology. One important solution to this problem is to develop high‐performance memory devices based on new architectures and functionalities such as resistive random access, ferroelectric random, and nonvolatile transistor memory. Another approach is to exploit multilevel storage functionality to enhance the capacity and reduce the memory space of individual memory units.^[^ ^25^, ^26^, ^27^ ^]^

Phototransistor is a photosensitive architecture in which incident light can modulate the channel charge carrier density, enabling the use of light as an additional terminal for optically controlling device operation along with the three conventional terminals. Thus, the phototransistor can be modulated not only via electrical stress but also through illumination, allowing it to serve as an additional channel for carrying out the programming function without mutual interference. More importantly, photonic memory is more competent than electric programming memory at achieving multilevel data storage with high discrepancy, as it avoids the drawback of destructive reading because the electric reading process is orthogonal to the light programming process.^[^ ^28^, ^29^, ^30^ ^]^ A high‐performance phototransistor memory will usually have a large memory window (the shift in threshold voltage between the ON and OFF states) and a high memory ratio (the difference in current between these two states, which distinguishes the data storage levels and reflects the reliability of the memory device). Although early studies focused primarily on photoinduced charge modulation in the transistor device channels and the application of this to photodetectors, the use of light illumination to assist in the charge storage effect further broadens the applicability of phototransistors by adding photoswitchability to the device's hysteretic characteristics. Such hysteresis allows for tuning at arbitrary wavelengths and illumination intensities, enabling photosensitive devices with specific photoresponsivities and photosensitivities and, most importantly, nonvolatile information storage. In this manner, phototransistors can convert signals provided by electric optics using amplification properties, and photomemory devices can store light information as electrical signals as building blocks for optical signal processing and photonic neuromorphic circuits. Moreover, combining the responses from electric fields and light irradiation enables advanced applications such as multibit data storage, light‐assisted encryption, dual‐mode operation with ambipolar memory effect, and photorecovery. Thus, the focus on organic phototransistors recently shifted to the exploitation of the efficacy of memory characteristics and synaptic behavior.^[^ ^31^, ^32^, ^33^ ^]^

Memory devices can be categorized as long‐term, short‐term, and sensor memory depending on their volatilities. An artificial synapse is regarded as a short‐term memory (STM) device with medium volatility, whereas a photodetector should not show hysteresis to optical or electrical stimuli and will produce a completely volatile signal after removing external stimuli. Generally, phototransistors with strong hysteresis are used as memory devices; in contrast, phototransistors with negligible hysteresis are good photodetectors. Artificial synapses lie in between memory and sensors: depending on the operational history, such devices will have medium volatility and hysteresis to external stimuli. It is worthwhile noting that with the growing development of neuromorphic computation, the design on efficient artificial synapses similar to those of biological synapses is of increasing importance.^[^ ^34^, ^35^ ^]^ A sensory synapse comprising photosynaptic transistors is more favorable than that of sensor integration comprising a photodetector and a nonvolatile memory in device miniaturization and emulating human perception systems executing processing and memory functionality with an ultralow energy consumption.^[^ ^36^, ^37^ ^]^

**Figure 1:**
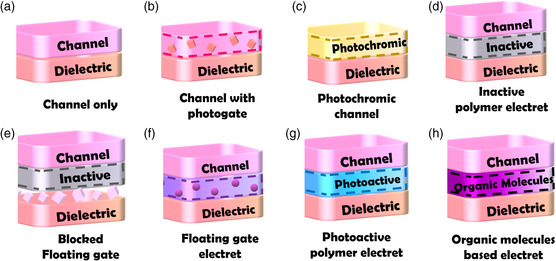
Device architectures of the phototransistor memory: a) channel‐only, b) channel with photogate, c) photochromic channel devices, and devices with d) inactive polymer, e) blocked floating gate, f) floating gate, g) photoactive polymer, and h) organic molecule‐based electrets.

**Figure 2:**
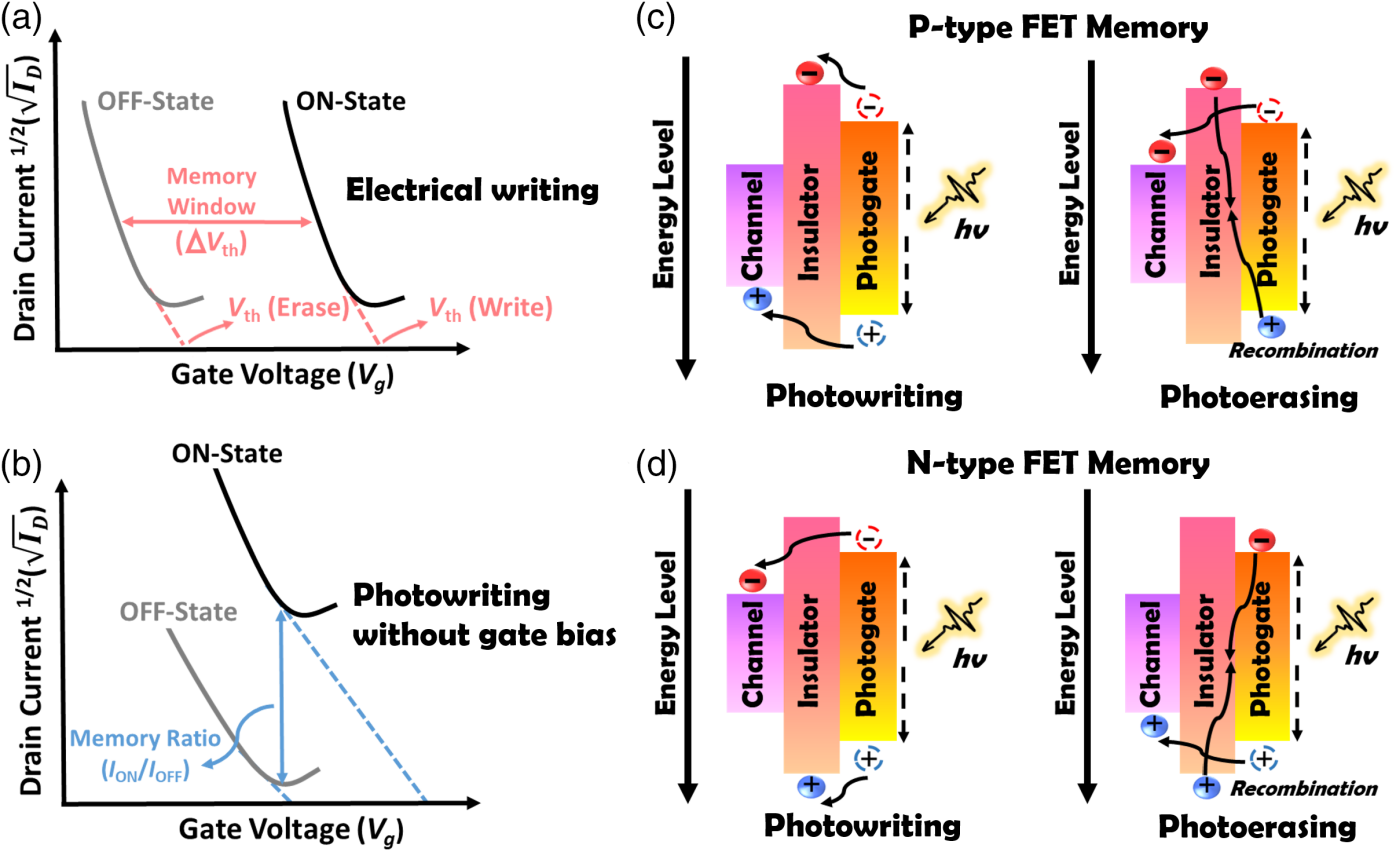
Memory characteristics of the device under a) electrical writing or b) photowriting without gate bias. Working mechanism of the c) p‐type or d) n‐type phototransistor memory under photowriting or photoerasing.

**Figure 3:**
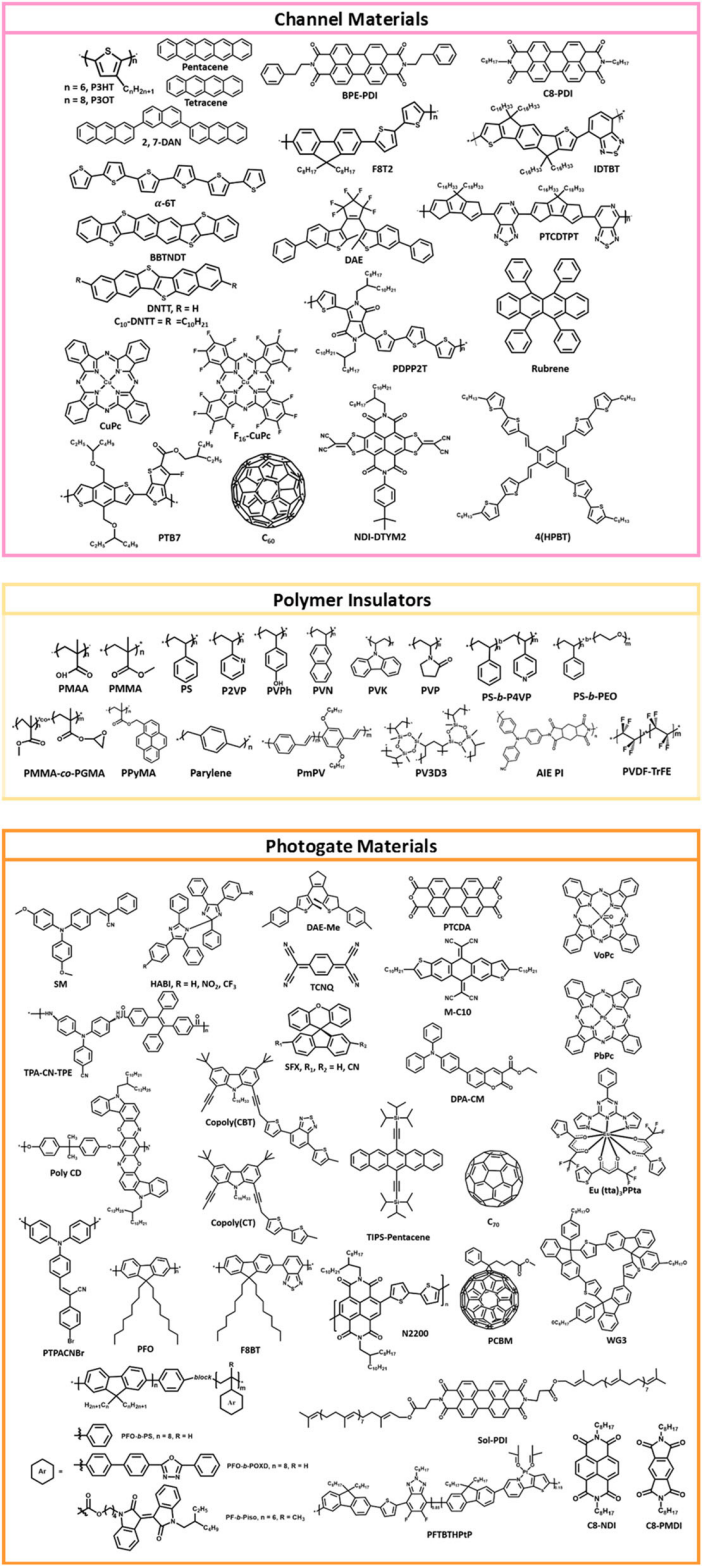
Chemical structures of the constituent materials in the reported phototransistor memory devices: channel materials (top), polymer insulators (middle), and photogate materials (down).

Although extensive research efforts have been dedicated to developing high‐performance phototransistors, electrically driven devices still predominate in data storage and processing applications. Still, phototransistors are being extensively investigated for their promising properties, which is our motivation here in depicting a full picture of the material selection and structural design of phototransistos. In this article, we present a review of the developments in phototransistors and the design of constituent materials. Starting from the nonvolatile memory application, device architectures can be categorized into single‐layer devices, including 1) channel‐only devices (**Figure** **1**a), 2) devices with channels and with photogates (Figure 1b), and 3) devices with photochromic channels (Figure 1c), and bilayer devices incorporating 4) inactive polymer electrets (Figure 1d), 5) polymer insulators with blocked floating gates (Figure 1e), 6) floating gate electrets (Figure 1f), 7) photoactive polymer electrets (Figure 1g), and 8) organic molecule‐based electrets (Figure 1h). In this article, the materials used in these device architectures are thoroughly discussed. After an introduction to phototransistor memory devices in Section 1, device architectures, performance characteristics, (**Figure** **2**a,b), and working principles (Figure 2c,d) are described in Section 2. In Section 3, material designs and the structure−performance relationships in different phototransistor memory device architectures are categorized and detailed. Other applications of phototransistors, including artificial synapses and photodetectors, are introduced in Section 4. Finally, the existing challenges and future outlook for phototransistors are presented in Section 5.

## 2 Device Architecture and Working Principle of Phototransistor

### 2.1 Working Principles to Nonvolatile Memory

A typical transistor‐type memory device comprises a charge storage layer in the form of, for instance, a floating gate or polymeric electret, inserted between a gate dielectric and a semiconducting channel. This structure allows each single transistor cell to possess at least two stable memory states that are controlled through external stimuli, such as electric fields applied from the gate bias or light illumination, to produce adjustable conductance states within the semiconducting channel, while precisely modulating the resulting source‐to‐drain current level and threshold voltage of the channel. Typical transfer characteristics (drain current (*I*_d_) versus gate voltage (*V*_g_)) are affected by the carrier concentration within a semiconducting channel located between source and drain electrodes, resulting in an amplified effect during voltage sweep from low‐to‐high electric fields. The threshold voltage (*V*_th_), which is the minimum gate voltage needed to create a conducting channel between the source and drain terminals, is determined by the intersection of the curves of *I*_d_^1/2^ versus *V*_g_ when *I*_d_ is zero. Thus, the extent of the built‐in electric field, which is determined by the density of trapped charge, can influence the resulting transfer curves, leading to shifts in the transfer curve at given polarities that define the current state. The storage of memorized information is enabled by the localization of polar charges in the bulk dielectric layer or between the interfaces of the gate dielectric and semiconductor dielectric channel. The variation in the threshold voltage during different operations is defined as the memory window. Another key parameter, the current switching memory ratio, is defined as the ratio of the maximum current in the high‐conductance state to the minimum current in the low‐conductance state when constructing a conventional binary digital signal of ‘‘0’'s (OFF) and ‘‘1’'s (ON) states. In general, electrical writing with a gate impetus will involve a respectably high‐threshold voltage shift with a resulting large memory window corresponding to the generation of a considerable number of charge traps (Δ*N* = *C*_i_ × *ΔV*_th_/*e*) in the bulk of the electret, where *C*_i_ is the capacitance of the electret, *e* is the elementary charge, and Δ*N* is the number of newly formed charge traps. In contrast, photowriting without a gate impetus involves a small change in the memory window but a significant difference in the current level of the ON state, resulting in a high device memory ratio induced by interfacial traps between the electret and semiconducting channel. Typically, the number of interfacial traps (*N*_t_) can be evaluated from the subthreshold swing (SS) of the transfer curve in the ON state using the relation *N*_t_ = (*C*_i_/*e* ^2^) × [(SS × *e*)/(*k*_B_ × *T *× ln10)−1], where *T* is the absolute temperature and *k*_B_ is the Boltzmann constant. Thus, photowriting without a gate impetus is more sensitive to interfacial properties, including the surface morphology and energy‐level adaptation between the semiconducting channel, photogate, and insulated matrix. Finally, photoassisted electrical writing involves a combinatory effect with both a large memory window and a large memory ratio, making this approach effective in obtaining multibit data storage with high discernibility. These characteristics and features are depicted in Figure 2a,b. Other important factors include memory retention and cycling endurance, which are used to assess the long‐term stability and reliability of the data storage performance of a device.

The basic architecture of a phototransistor memory is similar to that of an electrically driven transistor memory comprising a three‐terminal source, drain, gate, and an additional memory layer between the semiconducting channel and gate dielectric. The current level in a phototransistor memory can be optically or electrically gated. In a photowriting process, a number of excitons in the form of electron and hole pairs are generated when light with a photon energy equal to or greater than the bandgap energy of the organic semiconductor is absorbed, leading to an increase in the drain current. In a p‐type memory device, photogenerated holes are much more mobile than electrons and play a prominent role in increasing the current, whereas electrons are presumably immobilized at trapping sites immediately after their generation. The rate of photowriting is proportional to 1) the flux of photons with energies greater than the absorption edge of the organic semiconductor used and 2) the magnitude of the transverse electric field at the interface. Typically, photowriting without a gate bias renders lower‐energy defects near the interface. In contrast, pure electrical writing or photowriting with gate bias induces deeper‐level defects in the bulk, thereby enhancing the memory window and hysteresis behavior. In the reading process, the decay in the drain current exhibits nonexponential characteristics with two distinguishable decay rates: 1) an initial rapid relaxation owing to the recombination of closely packed holes and electrons and 2) a subsequent slower relaxation originating from deeply trapped carriers. In an electrical erasing process, gate bias is applied to the device, causing the carriers in the channel to attract and recombine with the trapped charges, thereby forming a built‐in electric field along the direction opposite to the applied voltage that returns the memory to the OFF state. It is understood that a shorter channel length results in a larger lateral electric field, which is beneficial for exciton dissociation. At the same time, a shorter channel length leads to faster carrier transport, thereby reducing carrier recombination in the channel. Because of their significance, channel dimensions are included in the listing of device characteristics in **Table** **1**, **2**, **3**. All of the factors discussed earlier enhance the photocurrent and alter memory performance.

**Table 1:** Reported material systems of the phototransistor memory comprising 1) channel‐only, 2) channel with photogate, or 3) photochromic channel configurations

Device	Photogate	Channel	Dielectric	*W*; *L* [μm]a)	Light [mW cm^−2^]b)	Modec)	*I*_on_/*I*_off_ d)	*ΔV*_th_ e)	Ref.
(i)	−−	F8T2	Parylene/SiO_2_	200; 40	0.01; 557 nm	EW; PE	−−	10 V	[42]
(i)	−−	4(HPBT)	SiO_2_	1500; 5	0.03; 436 nm	PW; EE	12; 100 s	15 V	[46]
(i)	−−	C8‐PDI	SiO_2_	2000; 40	250	PW; EE	10^5^; 10^4^ s	30 V	[48]
(i)	−−	NDI‐DTYM2	SiO_2_	2800; 10	107; white	PW; EE	10^4^; 8000 s	−−	[49]
(i)	−−	Rubrene	SiO_2_	60; 55	10; 460 nm	PW; EE	10^3^; 10^4^ s	25 V	[50]
(i)	−−	BBTNDT	SiO_2_	1440; 80	40; white	pEW; EE	10^6^; 10^4 ^s	60 V	[51]
(i)	−−	2,7‐DAN	SiO_2_	340; 40	0.65; white	EW; PE	−−	40 V	[52]
(ii)	CdSe@ZnSe QDs	P3HT	SiO_2_	1000; 10	2.8; white	PW; EE	3000; 8000 s	10 V	[54]
(ii)	NaYF4: Yb,Er@NaYF_4_	P3HT	SiO_2_	3000; 6	200; 980 nm	pEW; EE	100; 10^5 ^s	10 V	[55]
(ii)	MAPbBr_3_ QDs	P3HT nanofibers	SiO_2_	1500; 25	42.5; 405 nm	PW; EE	10^3^; 10^4 ^s	17 V	[56]
(ii)	CsPbBr_3_ QDs	PCDTPT	SiO_2_	−− f)	0.5; 300 nm	PW; EE	10^4^; 5 × 10^4^ s	67 V	[63]
(ii)	N2200	IDTBT	SiO_2_	1000; 30	0.1; 675 nm	PW; EE	5000; 10^4^ s	37 V	[64]
(iii)	−−	DAE	PMAA/ SiO_2_	400; 50	39; 365 nm	pEW; pEE	100	15 V	[65]
(iii)	*p*‐NO_2_‐HABI	PDPP4T	SiO_2_	−−	40; 365 nm	PW; TEg)	6800; 10^4^ s	−−	[66]
(iii)	DAE‐Me	P3HT	PVDF‐TrFE	10 000; 20	0.001; 310 nm	EW; PW	200; 10^6^ s	−−	[69]

a) *W*: channel width; *L*: channel length;

b) light intensity and wavelength;

c) EW: electrical writing; PW: photowriting; EE: electrical erasing; PE: photoerasing; pEW: photoassisted electrical writing; pEE: photoassisted electrical erasing;

d) *I*_on_/*I*_off_: memory ratio;

e) Δ*V*_th_: memory window;

f) vertical architecture in the transistor memory;

g) TE: thermal erasing.

**Table 2:** Reported material systems of the phototransistor memory with 4) inactive polymer, 5) blocked floating gate, or 6) floating gate electrets

Device	Electret	Channel	Dielectric	*W*; *L* [μm]a)	Light [mW cm^−2^]b)	Modec)	*I*_on_/*I*_off_ d)	Δ*V*_th_ e)	Ref.
(iv)	PS	Pentacene	SiO_2_	3000; 50	1; white	pEW; EE	10^6^; 10^4 ^s	35 V	[71]
(iv)	PS	DNTT	SiO_2_	2000; 100	20; 450 nm	pEW; EE	10^5^; 10^4^ s	170 V	[75]
(v)	PS/Au NPs	Pentacene	SiO_2_	750; 100	300; 530 nm	PW; EE	10^7^; 10^3^ s	40 V	[77]
(v)	c‐PVP/Au NPs	Graphene	Al_2_O_3_	900;200	1; 520 nm	PW; EE	3; 10^4^ s	−−	[78]
(v)	PV3D3	C_70_	PV3D3	1000; 200	3.8; white	pEW; EE	10^4^; 700 s	9 V	[80]
(vi)	PS‐*b*‐P4VP/Au NPs	F_16_‐CuPc	SiO_2_	1000; 100	74; white	EW; pEE	10^3^; 10^3 ^s	11 V	[83]
(vi)	PVPy/Au@Ag NRs	Pentacene	SiO_2_	1000; 50	10; 660 nm	EW; pEE	−−	20 V	[84]
(vi)	PVN/C_60_	Pentacene	SiO_2_	1000; 50	0.5; 350 nm	EW; PE	10^5^; 10^4^ s	21 V	[85]
(vi)	PVN/PCBM	Pentacene	SiO_2_	850; 100	0.17; 740 nm	pEW; EE	10^4^; 10^3^ s	13 V	[86]
(vi)	PMMA/DPA‐CM	Pentacene	SiO_2_	2000; 50	4.6; 365 nm	pEW; pEE	10^5^; 10^6^ s	12 V	[88]
(vi)	TPA‐SM/TPA‐PI	Pentacene	SiO_2_	1000; 50	0.72; 365 nm	pEW; pEE	10^5^; 10^4^ s	37 V	[89]
(vi)	PMMA/CsPbBr_3_ QDs	Pentacene	SiO_2_	3000; 100	93; 405 nm	PW; EE	10^3^	17 V	[90]
(vi)	Fluoroalkyl CdSe QDs	Pentacene	SiO_2_	1000; 50	1; 532 nm	EW; PE	10^4^; 10^4 ^s	20 V	[92]
(vi)	PMAA/PFTBTAPtP	Pentacene	SiO_2_	1000; 50	12.3; 530 nm	EW; PE	10^5^; 10^4 ^s	50 V	[93]
(vi)	PS/MAPbBr_3_ NCs	Pentacene	SiO_2_	1000; 50	71; 450 nm	PW; EE	10^4^; 7 × 10^6^ s	7 V	[94]
(vi)	PS‐*b*‐PEO/MAPbBr_3_ NCs	P3HT	SiO_2_	1000; 50	454; 520 nm	PW; EE	10^4^; 4 × 10^4^ s	−−	[96]
(vi)	PVPh/Cs_2_Pb(SCN)_2_Br_2_	Pentacene	SiO_2_	1000; 50	0.18; 450 nm	PW; EE	10^6^; 10^4 ^s	37 V	[97]
(vi)	P2VP/MAPbBr_3_ NCs	Pentacene	SiO_2_	1000; 50	10; 530 nm	PW; EE	10^5^; 10^4^ s	−−	[98]
(vi)	P2VP/MAPbBr_3_ NCs	BPE−PDI	SiO_2_	1000; 50	10; 530 nm (PW) 1.8; 254 nm (PE)	PW; PE	10^4^; 10^4^ s	14 V	[99]

a) *W*: channel width; *L*: channel length;

b) light intensity and wavelength;

c) EW: electrical writing; PW: photowriting; EE: electrical erasing; PE: photoerasing; pEW: photoassisted electrical writing; pEE: photoassisted electrical erasing;

d) *I*_on_/*I*_off_: memory ratio;

e) Δ*V*_th_: memory window.

**Table 3:** Reported material systems of the phototransistor memory with 7) photoactive polymer or 8) organic molecule‐based electrets

Device	Electret	Channel	Dielectric	*W*; *L* [μm]a)	Light [mW cm^−2^]b)	Modec)	*I*_on_/*I*_off_ d)	Δ*V*_th_ e)	Ref.
(vii)	PmPV	CNT	SiO_2_	50; 10	365 nm	pEW; EE	4; 5 × 10^4^ s	5 V	[100]
(vii)	TPA‐CN‐TPE	Pentacene	SiO_2_	1000; 50	0.72; 365 nm	pEW; pEE	10^4^; 10^4^ s	42 V	[101]
(vii)	Poly(CD)	Pentacene	SiO_2_	1000; 50	25; white	EW; PE	10^3^; 10^4^ s	82 V	[102]
(vii)	Copoly(CBT)	Pentacene	SiO_2_	1000; 50	25; white	EW; PE	10^3^; 10^4^ s	30 V	[103]
(vii)	PTPA‐CN	Pentacene	SiO_2_	1000; 50	5; 405 nm	pEW; EE	10^5^; 4 × 10^4^ s	15 V	[104]
(vii)	*β*‐PFO	Pentacene	SiO_2_	2000; 100	5; white	EW; PE	10^3^; 8 × 10^3^ s	57 V	[105]
(vii)	PFO	Pentacene	SiO_2_	1000; 50	25; 405 nm	EW; pEE	10^7^; 4 × 10^3^ s	76 V	[106]
(vii)	PS/PFO	Pentacene	SiO_2_	1500; 20	10; 405 nm	PW; EE	5 × 10^4^; 10^5^ s	20 V	[107]
(vii)	F8BT/PS	C_10_‐DNTT	SiO_2_	1000; 100	19.5; 455 nm	PW; EE	10^3^; 2 × 10^4^ s	14 V	[108]
(vii)	PFO‐*b*‐PS	Pentacene	SiO_2_	1000; 50	30; 405 nm	PW; EE	10^4^; 10^4^ s	21 V	[109]
(vii)	PFO‐*b*‐POXD	BPE−PDI	SiO_2_	1000; 50	1; 254 nm	EW; PE	10^5^; 10^4^ s	20 V	[110]
(vii)	PF‐*b*‐Piso	Pentacene	SiO_2_	1000; 50	30; 405 nm	PW; EE	10^6^; 10^4^ s	33 V	[111]
(vii)	PPyMA/TCNQ	Pentacene	SiO_2_	1000; 50	0.95; 365 nm	PW; EE	10^6^; 10^4^ s	34 V	[112]
(viii)	M‐C10	Pentacene	SiO_2_	−−	0.1; white	pEW; pEE	10^5^; 2 × 10^4^ s	10 V	[113]
(viii)	Sol‐PDI	BPE−PDI	SiO_2_	1000; 50	10; 530 nm	PW; EE	10^5^; 10^4 ^s	8 V	[114]
(viii)	C8‐NDI	BPE−PDI	SiO_2_	1000; 50	10; 405 nm	PW; EE	10^5^; 10^4^ s	6 V	[116]
(viii)	Eu(tta)_3_ppta	Pentacene	SiO_2_	1000; 50	0.5; 365 nm	pEW; pEE	10^4^	23 V	[117]
(viii)	DCNSFX	Pentacene	SiO_2_	1000; 50	1.8; white	EW; pEE	100; 10^4^ s	40 V	[118]
(viii)	C60/PTCDA	PbPc	ODTS‐PVA/SiO_2_	2000; 50	0.13; 405 nm	PW; EE	5 × 10^4^	69 V	[119]
(viii)	*p*‐6 P	VOPc	SiO_2_	200; 8	4.2; 365 nm	pEW; EE	10^5^; 5 × 10^3^ s	39 V	[120]
(viii)	WG_3_	Pentacene	SiO_2_	2000; 100	5; white	EW; PE	10^5^; 10^4^ s	45 V	[28]

a) *W*: channel width; *L*: channel length;

b) Light intensity and wavelength;

c) EW: electrical writing; PW: photowriting; EE: electrical erasing; PE: photoerasing; pEW: photoassisted electrical writing; pEE: photoassisted electrical erasing;

d) *I*_on_/*I*_off_: memory ratio;

d) Δ*V*_th_: memory window.

### 2.2 General Concepts of Phototransistors

A phototransistor can trap charge based on 1) intrinsic defects in the semiconducting channel, 2) at the interfaces between the channel/electret or electret/gate dielectric (usually, these are SiO_2_), or 3) inside the electret. The types of traps can be distinguished using multiexponential function fittings to the pulse response of a phototransistor. During light stimulating, both the rise and decay times should be limited by the charge‐trapping/detrapping process because charge generation and carrier drifting are much faster than them.^[^ ^38^ ^]^ The multiexponential decay of photocurrent suggests that long (deep)/short‐lived (shallow) charge traps are responsible for the observed time response and that a combination of multiple trap states with different lifetimes produces the observed overall temporal characteristics.^[^ ^39^, ^40^ ^]^ Organic semiconductors require more sophisticated designs in which intrinsic defects are introduced without sacrificing their charge transport capabilities. With regard to the shallow traps related to the gate dielectric, the common shallow traps on a thermally grown SiO_2_ dielectric surface includes 1) SiO_2_/Si interface trapped charges due to the interruption of the periodic Si lattice structure, 2) fixed immobile oxide charges near the SiO_2_/Si interface, 3) mobile ionic charges due to alkaline‐ion (e.g., Na^+^) contaminants that can be manipulated by bias‐temperature aging, 4) oxide‐trapped space charge associated with defects in SiO_2_, and 5) surface water and other chemiadsorbents in the form of Si−OH or other functional groups.^[^ ^41^ ^]^ The shallow traps related to SiO_2_ are easily removed and not reliable and controllable. Therefore, by introducing a photoactive electret below the channel, controllable photoinduced and field‐assisted charge trapping/detrapping can be utilized to enhance the charge‐trapping capability of a phototransistor. In addition, based on the aforementioned observations, photoprogramming without a gate impetus involves a small change in the threshold voltage shift but a significant difference in the current level, resulting in a high photosensivity induced by interfacial traps between the electret and semiconducting channel, and photoprogramming without a gate impetus is more sensitive to interfacial properties, including the surface morphology and energy‐level adaptation between the semiconducting channel, photogate, and insulated matrix.

Inserting a photoactive electret such as a floating gate or polymer electret into a phototransistor will significantly alter the photoresponse characteristics by strengthening the hysteresis and completely altering the preferences for dissociated charges. Incorporating a photoactive electret between the semiconducting channel and dielectric layer of a phototransistor can directly alter the dipole moment and internal capacitance and regulate the charge flow in the device. Thus, we will draw considerable attention to the rational design of specifications through the matching of energy levels and the tailoring of the morphology within the electret to ensure high device performance. Charge‐trapping polarity is directly determined by the adjacent energy levels of the highest occupied molecular orbital (HOMO) and lowest unoccupied molecular orbital (LUMO) levels, which serve as intuitive indicators of hole and electron migration and stabilization. Careful selection of the materials used to form a favorable energy‐level alignment is key to enhancing memory performance. It is worth noting that in a p‐type device, ΔHOMO between an insulated matrix and a channel should be large to prevent hole backtrapping, and a high ΔLUMO between an insulated matrix and a photogate is conducive to enhancing the stability of trapped electrons; in contrast, in an n‐type device, ΔLUMO between an insulated matrix and a channel should be large to prevent electron backtrapping, and a high ΔHOMO between an insulated matrix and a photogate is conducive to enhancing the stability of trapped holes, as illustrated in Figure 2c,d.

Morphology issues can be further divided into the bulk distribution of the photogate and the interfacial profile between layers. For example, a high‐performance floating gate electret usually relies on a strong correlation between the charge retention and distribution of photogates, while the interfacial profile can affect the charge transfer between channels and electrets, and a direct contact between the photogate and channel will result in a shortcut in which trapped charges are lost, leading to poor memory performance. Concisely, the design of photogate‐in‐electret is more competent than that of photogate‐in‐channel in nonvolatile memory applications. In contrast, the design of photogate‐in‐channel is revealed to be viable to achieve highly sensitive photodetectors and low‐energy‐consumption artificial synapses, having high volatilities and plenty of interfacial contacts between channels and photogates. It is understood that photoprogramming without a gate impetus is more sensitive to interfacial properties such as surface morphology and energy‐level adaptation between constituent materials. Therefore, we will also provide an in‐depth dissection of the interfacial characteristics of phototransistors.

## 3 Development of Phototransistor Memory

The typical strategy for obtaining a memory effect is to embed charge storage sites, such as metallic or semiconducting nanomaterials, or insert a polarizable polymer/organic electret between the semiconducting channel and gate−dielectric interfaces. During the writing/erasing processes, charges migrate back and forth in the active channel and through trapping sites under a programming/erasing voltage or specific light illumination. The trapped charges modulate the built‐in electric field in the device and produce a hysteresis effect that defines the discriminable memory states. There are several approaches to achieving memory characteristics in a phototransistor through the use of constituent materials that can be divided into channel materials (top), polymer insulators (middle), and photogate materials (down), as shown in **Figure** **3**. Table 1 summarizes the structural and characteristic parameters of 1) channel‐only, 2) channel‐with‐photogate, and 3) photochromic channel memory devices. Table 2 summarizes the characteristics of devices with 4) inactive polymer electrets, 5) polymer insulator‐blocked floating gates, and 6) floating gate electrets. Table 3 summarizes the characteristics of devices with 7) photoactive polymer‐ and 8) organic molecule‐based electrets.

### 3.1 Channel‐Only Devices

Salleo et al. first observed the phenomenon of photoinduced bias‐stress reversal in a transistor device fabricated from poly(dioctylfluorine‐*co*‐bithiophene) (F8T2), which was related to the optical absorption of F8T2.^[^ ^42^ ^]^ The bias‐stress effect essentially involves the removal of mobile carriers from the channel without affecting carrier mobility and the recovery of the bias‐stress effect from the recombination of photoinduced carriers or excitons with deeply trapped charges within the semiconductor and near the dielectric interface. Note that Salleo et al. postulated a potential trapping mechanism involving the pairing of two mobile holes to form a nonconducting bipolaron. Subsequently, Dutta et al. and Ujimoto et al. found similar phenomena involving gate‐controlled and photoinduced memory effects in field‐effect transistor (FET) devices with poly(3‐hexylthiophene) (P3HT) channels.^[^ ^43^, ^44^ ^]^ They found that, during the photoexcitation process, charges were generated throughout the channel with an intensity reflecting the light intensity and the local field. The resulting increased hole mobility played a significant role in the photoinduced charge transport, whereas the minor charges and electrons were localized immediately after being generated. The memory ratios of devices at the time of their investigations, however, were ≈10–100.

Various semiconducting materials have been investigated with the goal of improving memory performance. For example, Noh et al. utilized a p‐type semiconductor of α‐6T or pentacene as an FET device channel and obtained a preliminary memory ratio of 1300 by photowriting with 365 nm UV light.^[^ ^45^ ^]^ The memory effect they obtained was attributed to electron accumulation or trapping at the channel/dielectric interfaces with hydroxyl groups formed on the SiO_2_ surface. Cho et al. reported an enhanced memory ratio of 4,000 using *π*‐conjugated molecules of 4(HPBT) as the channel in an FET device.^[^ ^46^ ^]^ Their improved ratio was attributed to the ordered structure and star‐shaped architecture of 4(HPBT) and the memory relaxation was attributed to defects in its dielectric/channel interfaces, its bulk trap densities, and the diffusion rate of its photoinduced charges. In subsequent studies, a number of *π*‐conjugated molecules, including copper(II) phthalocyanine (CuPc),^[^ ^47^ ^]^ *N*,*N’*‐dioctyl‐3,4,9,10‐perylene tetracarboxylic diimide (C8‐PTCDI),^[^ ^48^ ^]^ NDI‐DTYM2,^[^ ^49^ ^]^ and rubrene,^[^ ^50^ ^]^ were applied as photoactive channels in phototransistor memory. Typically, the photowriting process is controlled by the light intensity and the applied gate field. Minor charges are trapped at the interface between the *π*‐conjugated semiconductor and the dielectric owing to the poor mobility of the *π*‐conjugated molecules, whereas major charges are much more mobile and play a prominent role in increasing the current. In addition to interfacial traps, bulk traps in the channel have also been investigated. For example, Li et al. postulated a memory effect with respect to the oxidation state of a rubrene single crystal in a rubrene‐based device through which photogenerated electrons could be trapped in oxidation defects.^[^ ^50^ ^]^

**Figure 4:**
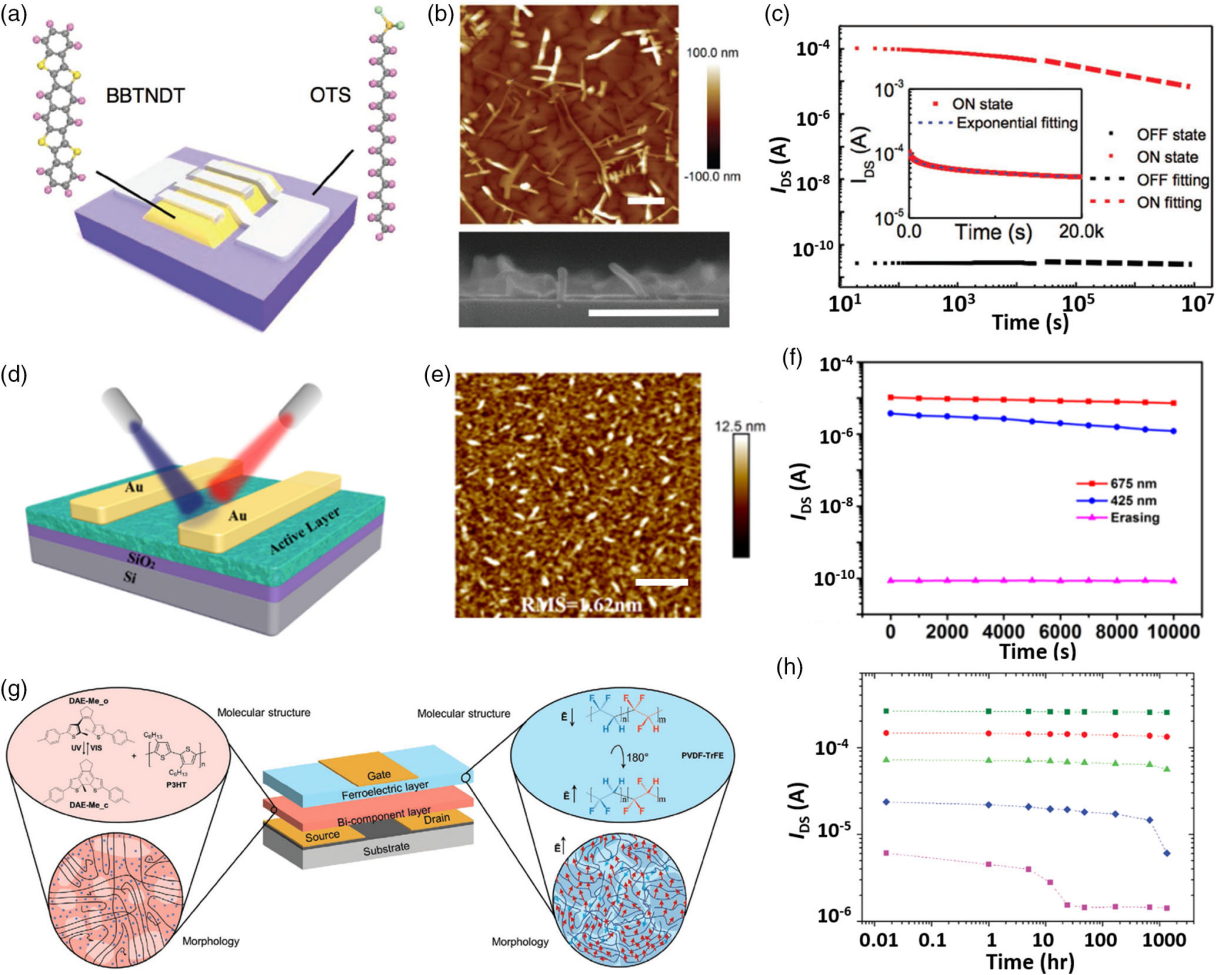
Phototransistor memory with a channel‐only architecture. a) Device architecture, b) AFM and SEM surface morphologies (the inset scale bars are 500 nm), and c) memory retention characteristics of the channel‐only device composed of BBTNDT with nanosprouts on the surface. Reproduced with permission.^[^ ^51^ ^]^ Copyright 2018, Wiley‐VCH. Phototransistor memory with the channel‐containing photogate. d) Device architecture, e) AFM surface morphologies (the inset scale bar is 1 μm), and f) memory retention characteristics of the memory device comprising polymer blends of IDTBT and N2200. Reproduced with permission.^[^ ^64^ ^]^ Copyright 2020, American Chemical Society. Phototransistor memory with a photochromic channel. g) Device architecture, working mechanism, and constituent materials and h) memory retention characteristics of the photochromic−ferroelectric organic transistor memory device comprising DAE‐Me, P3HT, and PVDF‐TrFE. Reproduced with permission.^[^ ^69^ ^]^ Copyright 2021, Wiley‐VCH.

The concept of introducing traps to the channel surface has recently been proposed. Implementing this approach would avoid the blockage of carrier transport by internal interfacial traps between the channel and dielectric. Pei et al. proposed a high‐performance phototransistor memory featuring a bis[1]benzothieno[2,3‐d;2’,3’‐d’]naphtho[2,3‐b;6,7‐b’]dithiephene (BBTNDT) channel with nanosprouts on the surface.^[^ ^51^ ^]^ **Figure** **4**a depicts their device architecture and Figure 4b shows the surface morphologies of BBTNDT with nanosprouts obtained through atomic force microscopy (AFM) and scanning electron microscopy (SEM). The nanosprouts are aligned along an orthogonal face‐on orientation relative to the BBTNDT film, which has an edge‐on orientation. The molecular misalignments at the interface between the nanosprouts and plain film behave as local defects, leading to uniformly distributed charge‐trapping sites on the channel. As a result, their BBTNDT‐based memory devices achieved an ultrahigh memory ratio of 10^6^ over 2 × 10^4^ s (Figure 4c). Later, Zheng et al. obtained photorecovery in FET memory devices by taking advantage of the intrinsic trap states caused by film inhomogeneity (nanosprouts) in 2,7‐DAN.^[^ ^52^ ^]^ However, because phototransistor memory with channel‐only conformations usually rely on gate bias to direct minor charges to the channel/dielectric interface during the photoprogramming and reading processes, destructive readout is commonly observed in this type of memory device.

### 3.2 Channels with Photogates

There are two viable approaches to improving the performance of phototransistor memory: 1) the photoresponse of a channel‐only device can be improved by incorporating photogates or photochromic materials in the channel and 2) the charge storage capability of a channel‐only device can be improved by introducing a photoinactive polymer electret. Depending on the structure of the channel, various types of photoactive materials can be incorporated into the semiconducting channel as a photogate. Through careful material selection, the photoresponse of the device can be enhanced and the photogate can be designed to serve as a charge‐trapping site through the formation of a favorable energy‐level alignment between it and the semiconducting channel. Chen et al. proposed the first phototransistor memory with this type of architecture based on a hybrid composite composed of P3HT and CdSe QDs; their device had a preliminary memory ratio of 100 over 200 s.^[^ ^53^ ^]^ Later, the same group designed core/shell QDs of CdSe@ZnSe to obtain an improved memory ratio of 2,700 over 8000 s.^[^ ^54^ ^]^ The heterojunction between the P3HT and QDs enhanced the separation of excitons, and the core/shell QDs served as trap centers that maintained the memory effect over long time periods even when the device was operated without gate voltage. Furthermore, the ZnSe shell layer between the CdSe core and P3HT produced an additional tunneling barrier that prevented electrons from tunneling back to P3HT, reducing the decrease in the drain current and providing a larger retention time than was obtainable in CdSe QD devices. In addition to QDs, upconversion nanocrystals (NCs) of Er^3+^/Yb^3+^‐codoped NaYF4 (NaYF4:Yb/Er@NaYF4) have been introduced to P3HT channels to enable upconverted phototransistor memory.^[^ ^55^ ^]^ In such devices, the IR absorption of upconversion NCs enables the emission of high‐energy photons at the expense of two or more low‐energy photons. Eventually, the high‐energy emission from upconversion materials is reabsorbed by the channel, ultimately improving device charge‐trapping efficiency and data‐storage level.

Recently, perovskite (PVSK) QDs have been shown to be promising photogate materials owing to their high photoluminescence quantum yields (PLQYs), ambient stabilities, long exciton diffusion lengths, and homogeneous size distributions. Ercan et al. proposed the first electrospun nanofiber‐only phototransistor memory comprising a polythiophene channel and a PVSK QD photogate. They achieved an acceptable memory ratio of 10^3^ over 10^4^ s, with high performance that could be attributed to the high aspect ratios of the nanofibers, regular 1D‐confined structures, and well‐dispersed PVSK QDs. Subsequently, a fiber‐based flexible memory device with a polyimide‐based substrate and a dielectric with excellent memory performance was shown to be useful in wearable electronics applications.^[^ ^56^ ^]^

In a conventional phototransistor, the photogenerated charges in a channel tend to recombine with each other due to the long channel length (usually several micrometers), producing a reduced separation efficiency. To overcome this obstacle, recently, a nascent approach by applying a vertical structure enables ultrashort channel length and the vertical pathway of charge transfer to suppress charge recombination. By adjusting the thickness of the channel, the channel length can be controlled to the submicrometer level or nanometer level. It is understood that a small channel length produces high conductance and low‐voltage operation of the device. Therefore, a vertical structure can improve the storage efficiency of nonvolatile memory,^[^ ^57^, ^58^ ^]^ reduce the power consumption in artificial synapses,^[^ ^59^, ^60^ ^]^ and be more conducive to realizing ultrafast photoresponse in photodetectors.^[^ ^61^, ^62^ ^]^ In addition, a vertical OFET is more favorable than a lateral OFET in large‐scale integration through crossbar stacking, and a vertical OFET is more tolerant of channel defects and cracks. This defect tolerance implies its potential in flexible device applications. Based on the aforementioned points of vertical structures, Yang et al. reported a multilevel vertical phototransistor memory comprising PCDTPT and PVSK QDs with a high memory ratio of 10^4^ over 5 × 10^4^ s.^[^ ^63^ ^]^ Their use of a vertical structure shortened the length of the charge transfer channel from tens of micrometers to the order of nanometers, which effectively reduced the trapped charge leakage and allowed for multilevel storage with high discrepancies and long‐term retention characteristics.

Advancements in the fabrication and design of high‐mobility *π*‐conjugated polymers have also improved the performance of photomemory. Lan et al. reported a photomemory comprising conjugated polymer blends of IDTBT and N2200.^[^ ^64^ ^]^ Figure 4d shows their device architecture. Figure 4e shows the AFM‐obtained surface morphology of an IDTBT film with 5% N2200, revealing the discrete domain of N2200 within IDTBT. The formation of a discontinuous N2200 domain with a high degree of roughness produced by a large number of trap centers improved the charge storage capability of the IDTBT channel. Their pristine IDTBT film exhibited a strong PL peak at 640 nm, which was quenched significantly after blending with N2200. Significant PL quenching was associated with efficient electron transfer from the LUMO of IDTBT to the LUMO of N2200, resulting in a device with a high memory ratio of 5 × 10^3^ over 10^4^ s that could be photowritten with 425 or 675 nm light, wavelengths consistent with the optical absorption of IDTBT (Figure 4f).

### 3.3 Photochromic Channels

Reliable multiresponsiveness can be achieved using utilizing photoswitchable (photochromic) molecules or exploiting the ferroelectricity of a gate insulator in the transistor memory. Photochromic materials can reversibly interconvert between two states following light irradiation. To take advantage of these properties, optically switchable transistors have been fabricated using photochromic channels, self‐assembled monolayers (SAMs), or dielectrics. As the former represents the most promising design with large conductance changes, in this section, we discuss the recent progress in the development of phototransistors with photochromic channels. The direct application of semiconducting photochromic materials is the simplest example of this approach. For example, Hayakawa et al. reported an optically switchable transistor comprising diarylethene (DAE) molecules, which serve as photochromic channels to perform semiconducting−insulating transitions through ring‐opening transformations.^[^ ^65^ ^]^ In addition, they introduced a thin layer of α‐6T to mitigate the intrinsic propensity of hole trapping in the DAE channel. Although the resulting device demonstrated a stable current contrast of 10^3^ over 10 endurance cycles, the relatively low mobility of the DAE molecules precluded a larger current contrast.

To overcome this obstacle, photochromic molecules have been blended into the semiconducting channel or directly tailored to semiconducting polymers to achieve high mobility in the photochromic channel. Based on the approach of blending, a photo‐/thermalresponsive transistor comprising poly(diketopyrrolopyrrole–bithiophene) (PDPP2T) and photochromic hexaarylbiimidazole (HABI) using photonic, electrical, and thermal programming, reading, and erasing signals, respectively, has been demonstrated.^[^ ^66^ ^]^ In this design, the HABI molecules fragment under UV light irradiation into pairs of triphenylimidazolyl radicals (TPIRs) that can thermally recombine to regenerate HABI molecules. The TRIRs present within the blended thin film following illumination can potentially interact with the charge defects in the PDPP2T channel to increase the carrier density. Impressively, the TPIRs do not affect the thin‐film crystallinity or morphology, enabling memory devices comprising PDPP2T and HABI to achieve stable memory ratios of 6800 over 10^4^ s. Although the processes used in fabricating these devices might not be currently practical, they effectively demonstrate orthogonal operation without mutual interference.

Directly tailoring the photochromic molecules in semiconducting polymers through structural engineering is considered to be an effective strategy for improving the compatibility and dispersion of the two components. Tian et al. reported a conjugated polymer containing azobenzene groups in its side chains (PDAZO) that could achieve optically switchable properties.^[^ ^67^ ^]^ The monomer with pendant azobenzene groups on its side chains was copolymerized with a monomer with typical branched alkyl side chains to form a photochromic conjugated polymer. Based on their theoretical calculations and X‐ray diffraction results, the changes in dipole moment and polymer configuration associated with the trans‐/cis‐photoisomerization of the azobenzene groups in PDAZO can affect the respective intrachain and interchain charge transport, enabling optically switchable transistor behavior. The device exhibited good photofatigue resistance with an ON/OFF ratio of 5 over five consecutive endurance cycles. More recently, the same group proposed a design for the simultaneous incorporation of two types of azobenzene groups into the side chains of conjugated polymers and demonstrated its applicability to photoassisted logic control.^[^ ^68^ ^]^ The monomers in their polymer were designed with asymmetric side chains comprising linear alkyl chains with pendant azobenzene groups or branched alkyl side chains, and two monomers with different types of pendant azobenzene groups were copolymerized to derive a photochromic conjugated polymer (POMPYA). It is worth noting that the trans‐/cis‐photoisomerization of meta‐ and para‐azobenzene groups can reversibly alter proximal interchain packing and thin‐film crystallinity, allowing POMPYA films to attain tristable semiconducting states corresponding closely to the conformations of the meta‐ and para‐azobenzene groups in the side chains.

Combining photochromic and ferroelectric gate dielectrics is considered to be a powerful tool for achieving multiresponsiveness in phototransistor memory. Carroli et al. reported the fabrication of a photochromic ferroelectric organic transistor memory^[^ ^69^ ^]^ by blending DAE‐Me with P3HT to form a photochromic channel and using PVDF‐TrFE as a ferroelectric polymer layer. PVDF‐TrFE offers several advantages over conventional polymer insulators; most importantly, it has relatively large fatigue‐free remnant polarization, short switching time, and high thermal stability (Figure 4g). The number of memory states in a ferroelectric memory cell can be increased using intermediate polarization states obtained by applying an external electric field with a magnitude slightly larger than the ferroelectric coercive field. Therefore, their memory device was capable of operating as a nonvolatile memory with 11‐bit memory storage capacity in a single device and had a high memory ratio of 200 over 10^6^ s. Figure 4h shows the data retention of their memory device as a function of the number of polarization cycles based on the readout current measured over several days. Note that their device was photoprogrammed at an ultralow light power of 1 μW cm^−2^ and short illumination duration of 3 ns at a frequency of 0.1 Hz.

### 3.4 Inactive Polymer Electrets

In the preceding section, we reviewed the functionality of incorporating photoactive materials into the channel. Here, we discuss the incorporation of inactive polymer electrets below the channel. Polymer electrets can be divided into photoactive and inactive types; here, we will review the functionality of inactive polymer electrets below the channel in storing photogenerated charge within the channel layer with the assistance of gate bias. A review of photoactive polymer electrets is presented in Section 3.7.

A channel‐only device can store charge based on intrinsic defects in the semiconducting channel or at the interface between the channel and gate dielectric (usually, these are SiO_2_). However, organic semiconductors require more sophisticated designs in which intrinsic defects are introduced without sacrificing their charge transport capabilities. Furthermore, the traps formed on the hydroxyl groups of the SiO_2_ surface tend to be shallow and easily removed. By introducing a polymer electret below the channel, the field‐assisted transfer of photoinduced charges from the channel to the polymer and localization of these charges by deep traps in the polymer can be obtained. The resulting immobilization of the transferred charge by the deep traps in the polymer produces a shift of the threshold voltage, which is conducive to enhancing the charge storage capability of a memory device.^[^ ^70^ ^]^

Based on this concept, Guo et al. reported a phototransistor memory comprising a pentacene or CuPc channel and PS or PMMA as an electret.^[^ ^71^ ^]^ The thin polymer layer is introduced to modify the SiO_2_ surface as the high‐energy‐electrets tunneling layer and the barrier layer to hold the low‐energy electrons tunneling back. The resulting device had a high memory ratio of 2 × 10^6^ over 2 × 10^4^ s for photowriting with white light at a gate voltage of 80 V. Subsequently, various combinations of pentacene channel and polymer electret, including PS,^[^ ^72^ ^]^ PMMA,^[^ ^72^, ^73^ ^]^ PMMA‐*co*‐PGMA,^[^ ^74^ ^]^ and PVK,^[^ ^75^ ^]^ were investigated and shown to have improved memory performance relative to their parent polymer electret‐free devices. For example, Ren et al. reported a phototransistor memory comprising a DNTT channel and PS as an electret.^[^ ^75^ ^]^ Illumination by blue light allowed photoinduced excitons to separate into holes and electrons. With the assistance of a positive gate bias, the electrons were injected from the DNTT channel into the polymer electret and the holes were transported through the channel. Notably, the memory device achieved a high memory ratio of 10^5^ over 10^6^ s. Because an in‐plane current occurred in the DNTT film without a PS electret or gate bias under the same blue light illumination, the long retention time was attributed not to a bulk effect of the DNTT but to the presence of the electron traps in the PS.

### 3.5 Polymer Insulator‐Blocked Floating Gates

Direct exposure of the photogate and semiconducting channels can result in the loss of trapped charge during reading. To remedy this, some studies have proposed the introduction of a floating gate below a thin polymer film. This thin film is usually inactive under applied light, allowing it to serve as a blocking layer for carriers in the channel and tunneling layer to enable charge trapping. In this device configuration, the floating gate is isolated from the channel itself and blocked by a polymer insulator, allowing the photosensitive function and semiconductor role to be designed individually and clearly separated. Illuminating a p‐type FET memory causes the photogeneration of excitons in the bulk of the channel film through the absorption of photons with higher energies than the semiconducting channel bandgap energy. By applying a positive gate bias, these excitons are separated into electrons and holes, and the separated electrons are injected and stored in the floating gates, whereas the holes are transported through the semiconducting channel, respectively. The photoinduced threshold voltage hysteresis originates from the accumulation of minority carriers at the interfaces, which in turn strongly depends on the photoirradiation intensity. Some results in which a blocked floating gate architecture was used to form NCs or nanoparticles (NPs) between the gate dielectric and polymer insulator have been reported. Using this approach, metal NPs, composed of Au^[^ ^76^, ^77^, ^78^ ^]^ or Ag,^[^ ^79^ ^]^ can be deposited onto the gate dielectric using e‐beam evaporation, sputtering, or thermal deposition to be embedded below a polymer tunneling layer composed of, for instance, PMMA,^[^ ^76^, ^79^ ^]^ PS,^[^ ^77^ ^]^ or crosslinked PVP.^[^ ^78^ ^]^ For example, Gao et al. reported a phototransistor memory comprising an embedded Au NP floating gate beneath an 11 nm‐thick PS layer with a high memory ratio of 10^7^ over 1000 s.^[^ ^77^ ^]^ They proposed that this memory window could be further enlarged by increasing the Au NP size and that using light with a photon energy equal to or greater than the HOMO−LUMO gap of the pentacene channel could induce the memory effect with the assistance of gate bias. Jang et al. later reported a phototransistor memory comprising embedded Au NPs as a floating gate under crosslinked PVP.^[^ ^78^ ^]^ Using a bilayered channel of pentacene and graphene, the photoresponsivity of the graphene channel could be enhanced by holes photogenerated in the top pentacene layer. They were able to fabricate an impressive flexible phototransistor memory on a poly(ethylene 2,6‐naphthalate) (PEN) substrate that demonstrated stable current retention and memory endurance to weak light.

Thermally deposited C_70_ has also been used as organic floating gate material. In one study, C_70_ was embedded via chemical vapor deposition as a pV3D3 layer, with C60 serving as the n‐type channel.^[^ ^80^ ^]^ The resulting device demonstrated a preliminary memory ratio of 10^4^ over 700 s. Hong et al. reported a phototransistor memory comprising an embedded PEDOT:PSS as a floating gate beneath a 20 nm‐thick Al_2_O_3_ layer in place of a polymer blocking layer. The Al_2_O_3_ layer was formed by atomic layer deposition and served as a blocking layer between the MoS_2_ channel and the floating gate. The resulting device demonstrated a high memory ratio of over 10^4^ over 2500 s under photoassisted writing and erasing.^[^ ^81^ ^]^ Han et al. reported a complementary inverter capable of UV‐assisted programming using Al_2_O_3_ as a blocking layer, CdSe/ZnS core−shell QDs as a floating gate, and pentacene/F16‐CuPc as the p/n channel.^[^ ^82^ ^]^ Electrons or holes could be confined in the cores of QDs because of band offsets under which the conduction band of the shell has a higher energy than that of the core and the valence band of the shell is lower than that of the core. The concept of using a CdSe/ZnS core−shell QD as a floating gate is similar to that mentioned in Section 3.2 of using a channel with a photogate. Notably, the inverter device demonstrated fast recovery under the application of switching voltage following illumination with UV light for 1 s.

### 3.6 Floating Gate Electrets

Conventional two‐step preparation methods for floating gates and tunneling layers are complex and not effective for large‐area processing. Degradation of device properties and variation in device‐to‐device performance are often observed owing to the use of multiple process steps of NP deposition, tunneling dielectric formation, and annealing, making the resulting devices more prone to the formation of interfacial defects. To overcome this obstacle, floating gate electrets with combined floating gates and polymer insulators have been developed. The floating gates used in these hybrid electrets can be prepared via a one‐step spin‐coating technique, avoiding the need to choose an orthogonal solvent for creating the tunneling layer to avoid damaging the floating gate. The resulting insulated polymers can effectively protect charges trapped within the floating gate and prevent charge leakage on both sides of the electret. In this section, we review phototransistor memory based on floating gate electrets, and the materials used as floating gates in a polymer matrix can be categorized into five types: 1) metal NPs, 2) organic molecules, 3) QDs, 4) polymer dots, and 5) PVSK NCs. With regard to inorganic nanomaterials, Leong et al. first fabricated a hybrid floating gate electret comprising PS‐*b*‐P4VP and Au NPs via in situ precipitation.^[^ ^83^ ^]^ The block copolymer (BCP)−NP system enabled homogeneous dispersion of the Au NPs and solution processability, which is especially suitable for low‐cost large‐area processing on flexible substrates. The resulting memory device had a preliminary memory ratio of 10^3^ over 10^3^ s under photoassisted writing/erasing with gate bias.

The localized surface plasmon resonance (LSPR) property of metal NPs can be utilized to construct materials with broad‐spectrum responsivity. Zhou et al. reported a hybrid floating gate electret in which the insulating and hydrophilic polymer PVP uniformly dispersed Au@Ag core−shell nanorods.^[^ ^84^ ^]^ The nanorods also possessed a plasmon resonance effect through which the plasmon resonance band could be manipulated by varying either the size of the Au cores or the thickness of the Ag shells to achieve a strong absorbance and a wide‐spectrum response range from the visible (the 500–600 nm range of the core−shell nanorods) to the near‐infrared (the 700–800 nm range of the Au cores) region. The resulting memory device had a preliminary memory window of 20 and 17 V after 10^4^ s under memory programming using photoassisted writing/erasing with gate bias.

**Figure 5:**
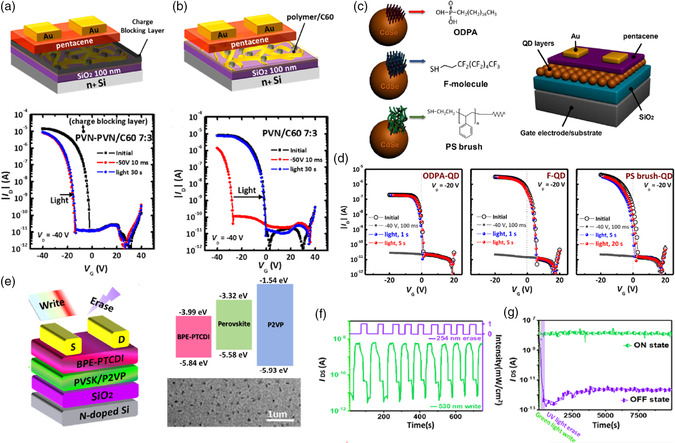
a,b) Device architecture (top) and transfer characteristics (bottom) after electrical writing and photoerasing of the memory device comprising a) blocked floating gate or b) floating gate electret of PVN and C_60_. Reproduced with permission.^[^ ^85^ ^]^ Copyright 2017, American Chemical Society. c) Chemical structure of the CdSe QDs and ligands and the architecture of phototransistor memory device comprising a pentacnee channel and the functionalized QDs as a floating gate electret. d) Transfer characteristics of the memory device under electrical writing and photoerasing. Reproduced with permission.^[^ ^92^ ^]^ Copyright 2018, American Chemical Society. e) Device architecture (left), energy‐level diagram of the constituent materials, and TEM morphology (right) of the PVSK/P2VP film. f) Endurance cycles and g) retention tests of the memory device under green light programming and ultraviolet‐C (UVC) light erasing at gate voltage of 0 V and drain voltage of 20 V. Reproduced with permission.^[^ ^99^ ^]^ Copyright 2021, American Chemical Society.

Various organic photoactive molecules, including C_60_,^[^ ^85^ ^]^ PCBM,^[^ ^86^ ^]^ TIPS−pentacene,^[^ ^87^ ^]^ DPA−CM,^[^ ^88^ ^]^ and triphenylamine‐based molecules,^[^ ^89^ ^]^ have been incorporated as photogates into insulated polymer matrices of PVN,^[^ ^85^, ^86^ ^]^ PMMA,^[^ ^87^, ^88^ ^]^ polyamide, or polyimide.^[^ ^89^ ^]^ Floating gate electrets comprising a photoactive molecule and insulated polymer can be used to program memory devices without gate bias and can enhance the charge‐trapping capability within the bulk of the electret. Phototransistor memory comprising C_60_ blocked by a PVN layer has no photoresponse to UV light (**Figure** **5**a), whereas phototransistor memory comprising a floating gate electret of C_60_ and PVN demonstrates enhanced memory windows in their transfer characteristics (Figure 5b),^[^ ^85^ ^]^ highlighting the efficacy of floating gate electrets in enhancing memory performance.

In addition to the insulated polymer, the photoresponse is highly dependent on the optical absorption of the incorporated organic molecules, and the insulated polymer is usually selected to form a compatible energy‐level alignment with the organic molecules. For example, Shiono et al. reported a top‐gate/bottom‐contact device configuration comprising P3HT as the channel, PMMA/TIPS−pentacene as the electret, and CYTOP as the dielectric.^[^ ^87^ ^]^ Their memory device could perform photoassisted electrical writing/erasing with blue/green/red light over a memory window of ≈25–30 V with a memory ratio of 10^2^–10^3^ over 3000 s. Huang et al. reported a hybrid floating gate comprising a triphenylamine‐based molecule (SM) and polyamide or polyimide.^[^ ^89^ ^]^ During UV‐assisted electrical writing, PL emissions produced by the photogating molecules are absorbed by top pentacene, producing photoinduced excitons that are transduced into holes and electrons. The electrons tunnel into the electret to achieve charge separation, and the organic molecules in the composite interlayers effectively generate excitons to primarily contribute to the elimination of trapped charges. In contrast, polyimides produce a wider bandgap than polyamides, potentially accommodating the improved trapping of charges. As a result, memory devices fabricated from polyimide and SM have demonstrated high memory ratios of 10^5^ over 10^4^ s, outperforming their polyamide and SM counterparts, which have modest memory ratios of 10^2^–10^3^.

Floating gate electrets comprising metal NPs or organic molecules usually rely on photoassisted electrical writing or erasing, a phenomenon potentially related to their weak photoresponses. A viable approach that incorporates high‐performance photogates can solve this problem, and inorganic QDs or PVSK NCs have been used as highly photoactive floating gates in phototransistor memory. With regard to inorganic QDs, the choice of polymer matrices and capping agents has a significant influence on their memory performance in floating gate electrets. For example, floating gate electrets comprising CsPbBr_3_ QDs in PMMA,^[^ ^90^ ^]^ or PVP^[^ ^91^ ^]^ matrices have been proposed. Lin et al. reported a series of n*‐*alkylmonoamines, dodecylamine, 1‐tetradecylamine, and 1‐hexadecylamine as surface‐capping agents to improve the stability of PVSK QDs.^[^ ^91^ ^]^ The higher melting points, lower steric hindrance, and stronger adhesion abilities of n‐alkylmonoamines relative to oleylamine provide optical and thermal stability. Notably, PVSK QDs have unique core−shell‐like structures in which the ligand layer acts as a natural tunneling region for the formation of a quantum well that effectively confines charges to the core. Jeong et al. fabricated CdSe QD‐only electrets capped with alkyl chains (ODPA‐QD), semifluoroalkyl chains (F‐QD), or PS brushes (PS‐QD), which are shown in Figure 5c.^[^ ^92^ ^]^ Figure 5d shows the transfer characteristics of one of these electrets following electrical writing and photoerasing. Their results suggested that the use of hydrophobic and small‐molecular surface ligands is effective at improving photoerasing performance. Notably, the formation of permanent dipole moments at the interface between the pentacene and fluorinated QD layers can modulate the energy level to enhance electron trapping.

In addition to the inorganic QDs, polymer dots have recently attracted considerable research interest as biosimulated photocatalysts in the energy conservation and renewable fuel industries because of compelling characteristics such as tailorable optoelectronic properties, amenability to structural modification, high water dispersibilities, and high stabilities under visible light‐driven processes. Polymer dots generally exist in the form of core−shell structures favorable to the isolation of trapped charges in a water‐soluble polymer matrix floating gate electret. For example, Liao et al. fabricated a series of polymer dots from donor−acceptor‐conjugated polymers composed of fluorene‐and thiophene‐flanked benzo[*d*]‐[1,2,3]triazole (PFTBTA) and a hydrophilic polymer of poly(ethylene glycol)‐functionalized PS.^[^ ^93^ ^]^ The polymer dots were dispersed in a water‐soluble PMAA matrix and used as an electret for phototransistor memory. By deliberately incorporating cycloplatinated pyridine into the polymer backbone (PFTBTAPtP), the memory device was capable of transitioning from volatile to nonvolatile memory as a result of the enhanced charge localization at the Pt sites. The resulting PFTBTAPtP memory device demonstrated a stable memory ratio of 10^5^ over 10^4^ s; in contrast, polymer dots without PFTBTA exhibit volatile memory ratios in the range of 10^5^−10^2^ over 10^4^ s.

PVSK NCs possess strong photoluminescence emission capabilities, low exciton binding energies, intense light‐harvesting capabilities, and long exciton lifetimes, making them promising candidates for use as photogates in floating gate electrets. Based on their compelling properties, PVSK NCs have been progressively developed for phototransistor memory applications. Chen et al. demonstrated the first hybrid composite composed of PVSK NCs and PS and its usefulness in phototransistor memory.^[^ ^94^ ^]^ The in situ formation of PVSK NCs in polymer matrices allows them to be evenly embedded into PS matrices to obtain acceptable photoresponsivities and ultrastable memory ratios of 10^4^ over 120 days. Later, PVPh, PMMA, and PMAA have also been investigated as insulated polymers in devices with PVSK NCs to evaluate their structure–performance relationships in phototransistor memory,^[^ ^95^ ^]^ with the results showing that the device photoresponse increases as the sizes of the embedded PVSK NCs decrease. Memory devices using PMMA as the insulated matrix were found to produce the smallest NCs (9–11 nm), giving them higher memory ratios (10^5^) than PS, PVPh, or PMAA‐based devices. In addition to evenly distributed PVSK NCs, the use of spatially addressable NCs in the insulated polymer matrix has been investigated. Chang et al. assessed the use of PS‐*b*‐PEO as an insulated matrix for PVSK NCs.^[^ ^96^ ^]^ By manipulating the interfaces and self‐assembled morphology of the floating gate electret, they were able to achieve an ultrafast charge transfer rate, efficiency, and memory response with an acceptable memory ratio of 10^2^ under 5 ms of light illumination.

The PVSK floating gate designs described earlier are all based on 3D NCs. Recently, 2D NCs have attracted extensive research interest owing to their excellent thermal and moisture durabilities and the ability of the layered 2D PVSK NC structure to naturally confines holes and electrons in quantum wells. Liao et al. proposed a floating gate electret of Cs_2_Pb(SCN)_2_Br_2_ in a PVPh matrix, producing a memory device with a pentacene channel with an ultrahigh memory ratio of 10^6^ over 10^4^ s under illumination by low‐intensity blue light (0.18 mW cm^−2^, 450 nm).^[^ ^97^ ^]^ This high photoresponsivity suggests that 2D PVSK NCs could improve the performance of phototransistor memory comprising floating gate electrets.

More recently, Yang et al. reported the use of P2VP as an insulated matrix for PVSK NCs.^[^ ^98^ ^]^ They found that, in the presence of P2VP, the sizes of the NCs could be restrained to the nanometer level of 7–9 nm, which is much smaller than that of 650–700 nm NC sizes obtained in the PS matrix. This improvement was attributed to the intense coordination between the pyridine in P2VP and lead ions in the PVSK through Lewis acid−base interactions. Owing to this NC size reduction, a memory fabricated from this floating gate and a pentacene channel could be programmed with a shorter illumination time of 5 s to achieve a high memory ratio of 10^4^ over 10^4^ s. In contrast, a reference device composed of PS and PVSK NCs required 60 s of light illumination to achieve a memory ratio of 10^2^. Subsequently, a fully light‐driven memory device was fabricated using a PVSK/P2VP floating gate electret with a BPE−PDI channel. Figure 5e shows the device architecture, energy‐level diagram, and tunneling electron microscope (TEM) morphology of the resulting PVSK/P2VP film. The memory device was photowritten with visible light for 20 s and then photoerased with UV light for 1 s to achieve a high memory ratio of 10^4^ over 10^4^ s.^[^ ^99^ ^]^ Figure 5h,i shows the memory endurance cycles and retention test results of a memory device with green light programming and UV light erasing at a gate voltage of 0 V and drain voltage of 20 V. Notably, this is a gate‐free and flexible memory device that uses only two terminals to perform both photowriting and erasing. These gate‐free, two‐terminal characteristics with carrier transport in the lateral direction are unique and compatible with flexible electronics. Impressively, the flexible memory device has a stable memory ratio of 10^3^ after 1000 bending cycles and at least ten consecutive endurance cycles. This effort was the first attempt to develop a fully light‐driven memory device without the assistance of gate bias and constitutes a proof of concept that warrants further investigation. Overall, with the rapid development of floating gate electrets, it is believed that floating gate electrets with polymer dots, inorganic QDs, and PVSK NCs will contribute to the further advancement of high‐performance phototransistor memory.

### 3.7 Photoactive Polymer Electrets

Photoactive polymer electrets possess bulk homogeneity, acceptable mechanical tolerances, and more diversified structural designs than hybrid composites, enabling direct investigation of their interactions with channels. Star et al. reported the first phototransistor memory using a functional assembly of the photoactive polymer of PmPV as an electret and a channel composed of carbon nanotubes (CNTs). The polymer layer converted photons to electric charges and then stored them and the CNTs transported the carriers. The resulting device had a preliminary memory ratio of 4 over 6 × 10^4^ s.^[^ ^100^ ^]^ Following this study, there was a long gap in the literature until very recently, when a number of photoactive polymer electrets including 1) photoluminescent polymers, 2) conjugated/insulated polymer blends, 3) conjugated BCPs, and 4) supramolecules were examined.

Photoluminescent polymers can be categorized as aggregation‐enhanced emission (AEE), aggregation‐caused quenching (ACQ), or aggregation‐induced emission (AIE) polymers. Cheng et al. introduced an electret composed of an AEE‐active polyamide (TPA‐CN‐TPE) that emitted strong green light under UV irradiation^[^ ^101^ ^]^ that could be subsequently absorbed by a pentacene channel. The photoinduced excitons in the channel could be separated with the assistance of gate bias, leading to significant shifts in the threshold voltage that could be considered photowriting. Their memory device could be erased by applying a reverse gate bias to achieve a high memory ratio of 10^4^ over 10^4^ s. However, an AEE‐inactive aromatic polyimide (TPA‐PIS) film exhibited very weak luminescence because it had a much stronger intramolecular charge transfer effect than that of TAP‐CN‐TPE. Later, Chen et al. reported an ACQ‐active polymer electret of poly (carbazole‐dioxazine) (polyCD). With its strong hole‐trapping capability, an ultrahigh memory window of 82 V could be achieved by electrically writing the polyCD‐based device.^[^ ^102^ ^]^ In the photoerasing process, the polyCDs efficiently generated excitons under light illumination to eliminate the trapped charges in the electret. They subsequently reported a conjugated ACQ‐active polymer (copoly(CBT)) electret in a phototransistor memory, producing a memory device with a stable memory ratio of 10^3^ over 10^4^ s.^[^ ^103^ ^]^ It is understood that introducing donor−acceptor systems is a good strategy for overcoming the Coulombic forces between electron and hole pairs to enhance the charge separation efficiency. In addition, *π*‐conjugated polymers can effectively facilitate the dissociation of electron−hole pairs through charge delocalization, particularly when the donor and acceptor moieties are highly twisted. Thus, copoly(CBT) with a twisted donor−acceptor‐conjugated structure can achieve ambipolar charge trapping and efficient photoerasing capability.

**Figure 6:**
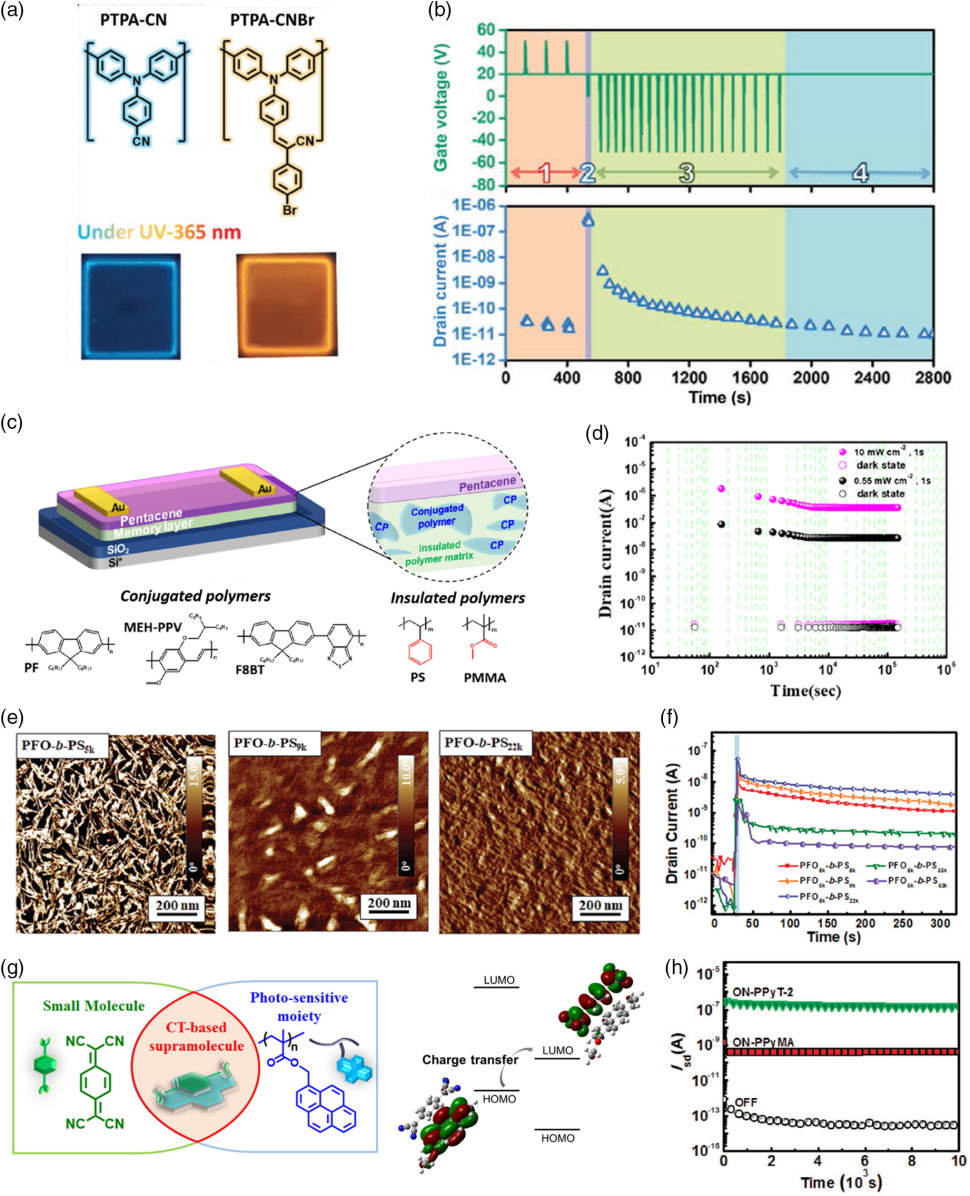
a) Chemical structures of the AIE‐active and photoluminescent polymers of PTPA‐CN and PTPA‐CNBr. b) Transient characteristics of the memory device comprising PTPA‐CN (flash type) under photowriting and electrical erasing by applying consecutive gate impetus. Reproduced with permission.^[^ ^104^ ^]^ Copyright 2021, Wiley‐VCH. Phototransistor memory with conjugated polymer blends as electrets. c) Device architecture and chemical structures of the conjugated polymers and insulated polymers in the polymer blend electrets. d) Retention tests of the phototransistor memory comprising PFO/PS polymer blends as an electret and a pentacene channel. Reproduced with permission.^[^ ^107^ ^]^ Copyright 2019, American Chemical Society. Phototransistor memory with conjugated BCP as an electret. e) AFM surface morphologies of PFO‐*b*‐PS with varied composition. f) Transient characteristics of the phototransistor memory comprising PFO‐*b*‐PS as an electret and a pentacene channel. Reproduced with permission.^[^ ^109^ ^]^ Copyright 2021, Royal Society of Chemistry. g) Chemical structures of PPyMA and TCNQ and the orbital distribution of their charge transfer complex. h) Retention test of the phototransistor memory with charge transfer supramolecular electrets. Reproduced with permission.^[^ ^112^ ^]^ Copyright 2021, Wiley‐VCH.

Photoluminescent polymers have been widely used as electrets in phototransistor memory devices with electrical writing/photoerasing functionalities. Recently, Ke et al. developed a series of AIE‐active poly(triphenylamine) (PTPA) polymers with finely tuned electron‐accepting moieties to enable photowriting/electrical erasing functionality in phototransistor memory.^[^ ^104^ ^]^ The chemical structures of these PTPA polymers are shown in **Figure** **6**a. After simultaneous excitation without application of an electric field, their AIE‐active conjugated donor–acceptor polymers underwent interlayer charge recombination and pronounced photowriting with a high memory ratio of 10^5^ over 4 × 10^4^ s. Notably, by twisting the dihedral angle between the donor and acceptor moieties, they could obtain tunable behavior from flash‐type (Figure 6b) and write‐once‐read‐many (WORM)‐type memory. Ling et al. reported the use of poly(9,9‐di‐n‐octylfluorenyl‐2,7‐diyl) (PFO) as a photoluminescent polymer electret for phototransistor memory.^[^ ^105^ ^]^ They found that PFO with β‐conformation is beneficial for electron trapping, thereby enhancing the memory stability. The resulting memory device achieved an acceptable memory ratio of 1000 over 8000 s under electrical writing and photoerasing processes with white light. The same approach was adopted by Chen et al., revealing an improved memory ratio of 10^7^ over 4,000 s under electrical writing and photoassisted electrical erasing processes with 405 nm light.^[^ ^106^ ^]^ However, the semiconducting nature of PFO deteriorates the stability of trapped charges, and destructive readout with gate bias is necessary to apply to operate these devices.

A sophisticated structure design and synthesis are necessary to produce a favorable polymer electret for use in phototransistor memory, making it of significant interest to develop a facile approach to producing high‐performance polymer electrets. In contrast, it is of great importance to overcoming the charge leakage in conjugated polymer electrets. With regard to the previously mentioned problems, our group systematically investigated a series of conjugated/insulated polymer blends as photoactive polymer electrets for phototransistor memory with multilevel photoresponse.^[^ ^107^ ^]^ The constituent conjugated polymers included PFO, poly[2‐methoxy‐5‐(2‐ethylhexyloxy)‐1,4‐phenylenevinylene] (MEH‐PPV), and poly[(9,9‐di‐n‐octylfluorenyl‐2,7‐diyl)‐*alt*‐(benzo[2,1,3]thiadiazol‐4,8‐diyl)] (F8BT); the constituent insulated polymers, including PS and PMMA, and their chemical structures and device architectures are shown in Figure 6c. We found that photogenerated electrons transferred to the proximate insulated polymers, and photogenerated holes were transduced to the pentacene channel to achieve photoinduced electron trapping in the photowriting process. To achieve electrical erasing, holes were injected from the pentacene channel to recombine with the trapped electrons. The high contact area between the conjugated and insulated polymers and the discrete distribution of the conjugated polymer resulted in an acceptable memory ratio of 5 × 10^4^ over 10^5^ s (Figure 6d). Later, Xu et al. applied a conjugated/insulated polymer blend of F8BT/PMMA into a three‐level phototransistor memory^[^ ^108^ ^]^ in which the memory states were defined using gate bias to obtain 0–1 state transition and gate bias with light illumination to obtain 1–2 state transition. The memory device had exceptional memory ratios of 10^3^ (0–2 state) and 10^2^ (0–1 state) over 2 × 10^4^ s.

It is worth noting that when using a BCP comprising conjugated/insulated polymers, severe phase separations observed in the conjugated/insulated polymer blends that hamper the performance of a phototransistor memory can be avoided. BCPs of PFO and PS (PFO‐*b*‐PS) introduced by our group as a polymer electret for phototransistor memory had a well‐dispersed and microphase‐separated morphology that stabilized the trapped electrons at the interface of the PFO and PS domains.^[^ ^109^ ^]^ As shown in Figure 6e, PFO‐*b*‐PS_22k_, which has an enhanced PS content, exhibits a smoother surface than PFO‐*b*‐PS_5k_, which has a fiber‐like structure with a low PS content. In addition to the morphological changes, PFO became β‐conformation as incorporated in BCPs, thereby improving the stability of trapped electrons. By optimizing the PS content of a memory device, it is possible to achieve a more stable photoresponse and memory window, as shown in Figure 6f. Notably, a memory device composed of PFO‐*b*‐PS_22k_ could achieve a high memory ratio of 10^4^ over 10^4^ s. Subsequently, BCPs composed of PFO and poly (vinylphenyl oxadiazole) (POXD) were used as electrets to investigate the influence of the donor–acceptor effect on memory performance.^[^ ^110^ ^]^ A memory device composed of PFO‐*b*‐POXD and BPE−PDI had an efficient electrical writing/photoerasing behavior with a high memory ratio of 10^5^ over 10^4^ s under illumination by 254 nm light with a low intensity of 1 mW cm^−2^. Recently, a panchromatic memory device using pentacene as a channel and BCPs composed of PF and poly (pendant isoindigo) (Piso) as an electret^[^ ^111^ ^]^ was demonstrated. Complementing the photoresponsivity of the PF to blue light, the Piso served as an insulated coil and a UV‐active polymer, allowing the memory device to respond to UVC (254 nm) and UVA (365 nm) light with memory ratios of 10^3^ and 10^4^, respectively. Notably, the photoinduced electrons in the pentacene channel could be transduced into the BCP electret without the assistance of a gate bias, which allowed the memory device to achieve memory ratios of 10^4^ and 10^2^ for green and red light, respectively. These results indicate that trimming electron‐donating/accepting groups in polymer electrets not only modulates their energy levels and optical absorption but also regulates the polarity and storage of the charges trapped within them.

Very recently, we were able to confirm highly efficient photoinduced recovery in phototransistor memory using a charge transfer supramolecular electret. The supramolecular electret, which was composed of poly(1‐pyrenemethyl methacrylate) (PPyMA) and 7,7,8,8‐tetracyanoquinodimethane (TCNQ), demonstrated electrical‐/optical‐induced memory bistability owing to the formation of numerous charge transfer complexes within it.^[^ ^112^ ^]^ Figure 6g shows the chemical structures of PPyMA and TCNQ and the orbital distribution of their charge transfer complexes. The favorable molecular association and dispersion between pyrene and TCNQ allowed for a high memory ratio of 10^6^ over 10^4^ s. The results of memory retention testing of the phototransistor memory are shown in Figure 6h. Testing of a reference device with an n‐type channel of BPE−PDI instead of a p‐type channel of pentacene revealed no field‐effect behavior in the device, demonstrating the necessity of forming unipolar charge transport in a memory device with a charge transfer supramolecular electret.

### 3.8 Organic Molecule‐Based Electrets

Small molecules have a number of advantages over photoactive polymers in that 1) they are easy to purify and have 2) batch‐to‐batch reproducibility, 3) well‐defined molecular structures, and 4) ordered intermolecular packings. Organic molecules designed for charge storage are also appealing as nonvolatile memory materials but lag far behind other molecules in terms of research on forming charge transport channels. Herein, we tried to summarize the recent progress in the phototransistor memory comprising an organic molecule‐based electret, and the recent studies on phototransistor memory using organic molecule‐based electrets can be categorized into 1) the formation of an ordered charge blocking layer between an organic molecule‐based electret and the channel and 2) the formation of a heterojunction structure between the organic molecule‐based electret and channel.

**Figure 7:**
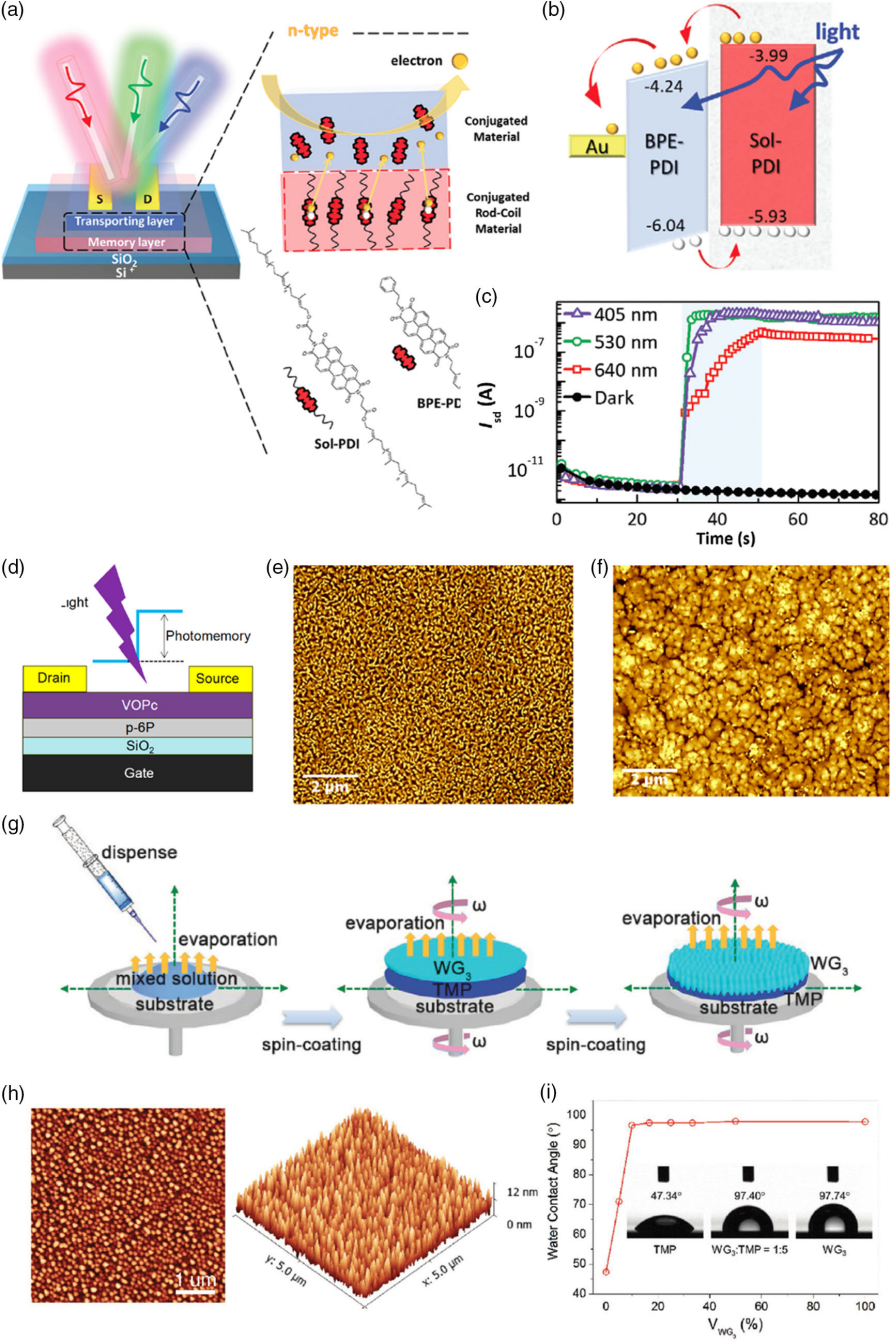
a) Device architecture and chemical structure of sol−PDI, b) energy‐level diagram and working mechanism, and c) transient characteristics of the phototransistor memory comprising rod–coil molecule as an electret and a BPE−PDI channel. Reproduced with permission.^[^ ^114^ ^]^ Copyright 2020, Wiley‐VCH. d) Device architecture and AFM surface morphologies of e) VOPc and f) p‐6P/VOPc bilayered film. Reproduced with permission.^[^ ^120^ ^]^ Copyright 2017, American Chemical Society. g) Preparation procedure of the nanostructured WG3 film. h) AFM topographies and i) water contact angle of the nanostructured WG3 surface. Reproduced with permission.^[^ ^28^ ^]^ Copyright 2018, Wiley‐VCH.

In terms of the first strategy, Zhang et al. first reported an organic molecule‐based electret of 2,2′‐(anthra[2,3‐b:6,7‐b′]dithiophene‐5,11‐diylidene)dimalononitrile with two decyl side chains (M‐C10).^[^ ^113^ ^]^ The long alkyl side chains facilitated the self‐organization of the M‐C10 layer, following thermal annealing, which produced a layer‐by‐layer motif that could isolate pentacene/M‐C10 interfaces, serving as an intrinsic tunneling area with excellent charge retention capability. Based on these characteristics, an enhanced transistor performance along with a prolonged charge retention time was achieved, with a memory ratio of 10^5^ over 2 × 10^4^ s. This performance was much better than that of memory with nonorganized M‐C10 and a reference molecule without the alkyl side chain (M‐C0), which had memory ratios of 10^5^ and 10^3^, respectively. A similar strategy was utilized by our group when using conjugated rod–coil materials to fabricate a phototransistor memory electret.^[^ ^114^ ^]^ In the phototransistor memory, a solanesol‐functionalized perylenediimide (Sol−PDI) was applied as the electret and BPE−PDI was used to fabricate the channel. The device architecture and the chemical structures of Sol−PDI and BPE−PDI are shown in **Figure** **7**a and the energy‐level diagram and working mechanism are shown in Figure 7b. By uniting a conjugated core in the channel and electret layers, barrier‐free interfaces between the electret and channel were successfully achieved. Furthermore, the self‐assembled behavior induced by thermal annealing defined the trapping sites and tunneling regions in the conjugated cores and side chains, respectively, enabling a fast response with an excellent memory ratio of 10^5^ over 10^4^ s and a high sensitivity to multiband light illumination (Figure 7c). Subsequently, an image‐sensing system based on the combination of p‐ and n‐type rod−coil molecules with an ultrafast photoresponse (<300 ms) and low‐power consumption was demonstrated.^[^ ^115^ ^]^ In addition to the rod–coil molecules, Lin et al. reported the use of rod‐like molecules of alkylated rylenediimides of 2,7‐dioctylbenzo[*lmn*][3,8]phenanthroline‐1,3,6,8(2*H*,7*H*)‐tetraone (C8‐NDI) and pyromellitic diimide of 2,6‐dioctylpyrrolo[3,4‐f]isoindole‐1,3,5,7(2*H*,6*H*)‐tetraone (C8‐PMDI) as phototransistor memory electrets.^[^ ^116^ ^]^ The rylenediimide films were thermally annealed at temperatures above their phase‐transition points to form liquid crystals, and homotropically aligned films were obtained by cooling from the liquid‐crystalline phases. The resulting rylenediimide films with 3D‐ordered smectic layer structures and brickwork stackings constituted effective phototransistor memory electrets, and a memory device obtained by combining them with an n‐type channel of BPE−PDI demonstrated an acceptable memory ratio of 10^5^ over 10^4^ s.

A number of studies have attempted to fabricate heterojunction structures between organic molecule‐based electrets and channels. Zhuang et al. developed a phototransistor memory using a lanthanide complex (Eu(tta)_3_ppta) as an electret.^[^ ^117^ ^]^ The luminescent complex of Eu(tta)_3_ppta emitted intense red light upon UV light excitation and served as a trapping element for holes injected from the pentacene channel. However, a memory device fabricated from the Eu(tta)_3_ppta electret and a pentacene channel required gate bias in both the writing and erasing processes, which meant that UV light could play only an auxiliary role in its programming. Sun et al. reported a series of spiro[fluorine‐9,9’‐xanthene] (SFX) derivatives as phototransistor memory electrets.^[^ ^118^ ^]^ By tuning their side groups, the molecular interactions, dipole moments, and memory characteristics of the SFXs could be modulated. In organic‐molecule electrets, the intrinsic dipole moment of the organic molecule, rather than charge dissipation, might play a vital role in the charge transfer between the electret and the channel. In addition, cruciform spiroarene demonstrates a typical supramolecular steric hindrance, and the large dihedral angle of its constituent donor–acceptor moieties might encourage charge trapping at heterojunction interfaces. Notably, the high dipole moment of dicyano‐functionalized SFX (DCNSFX, 5.5 D), which exceeds that of SFX (1.0 D), is conducive to hole tunneling in both electrical writing and photoerasing. DCNSFX possesses a larger memory window than SFX (39 as opposed to 31 V), and memory devices composed of DCNSFX have achieved memory ratios of 10^2^ over 10^4^ s.

Given that inferior exciton dissociation is a potential obstacle to photon absorption conversion into free charge carriers in single‐panchromatic materials, a multimaterial compound strategy is the preferred option because, and in addition to enabling broadband absorption, it increases exciton dissociation efficiency owing to the use of a heterojunction structure. Du et al. reported a multimaterial phototransistor memory comprising lead phthalocyanine (PbPc, near infrared (NIR) red light), perylene dianhydride (PTCDA, green−blue light), and fullerene (C_60_, purple UV light).^[^ ^119^ ^]^ The effectiveness of its heterojunction structure was reflected in its panchromatic photoresponse with a high memory window of 69 V under 405 nm light programming. However, the lack of an insulated medium for charge storage resulted in poor retention in the memory window of 15 V after 10^4^ s. Qian et al. reported a phototransistor memory with a vanadyl‐phthalocyanine (VOPc) channel on a p‐6P thin film.^[^ ^120^ ^]^ The device architecture is shown in Figure 7d. p‐6P is a very effective interface modifier for enhancing the light absorption and electron‐trapping capability of a heterojunction at its interface. As shown in Figure 7e,f, a more segregated morphology can be observed in the p‐6P/VOPc film than in the VOPc film, and the heterojunction between VOPc and p‐6P results in superior photomemory characteristics with a memory ratio of 10^5^ over 5000 s. However, the lack of an insulated medium for charge storage requires the use of a destructive readout with gate bias.

While narrow‐bandgap materials can lose trapped charge, resulting in data retention degradation, wide‐bandgap semiconductors have high potential barriers, making them ideal for preserving trapped charges and, therefore, of potential use in memory devices. Li et al. fabricated isolated and ordered nanostructure arrays from the blending of small wide‐bandgap molecules with trimethylolpropane followed by phase separation via spin coating.^[^ ^28^ ^]^ Their fabrication procedure and the resulting AFM morphologies and surface hydrophobicity are shown in Figure 7g–i, respectively. Their device had a high memory ratio of 10^5^ over 10^4^ s, which was enhanced by the improved charge‐trapping efficiency and the increased contact area between WG_3_ and the pentacene channel. The isolated nanostructure suppressed the lateral diffusion of the trapped holes among the nanocolumns, giving the memory device high stability and reliability. In addition, the electron traps were primarily located in the crystal grain boundaries, as indicated by the in situ conductive AFM under the application of a 365 nm light pulse. Despite the variety of molecule‐based electret designs, most require a gate voltage to program or read the memory device; to eliminate the application of gate bias in memory programming, isolated nanostructures or layered structures should be further developed.

Collectively, phototransistor memory devices comprising 1) channel‐only, 2) channel with photogate, or 3) photochromic channel configurations are favorable for fast response applications but usually require gate bias to program or even read the device. However, this design is revealed to be viable to achieve highly sensitive photodetectors and low‐energy‐consumption artificial synapses, having high volatilities and plenty of interfacial contacts between channels and photogates. Next, by introducing a polymer electret below the channel, it is possible to achieve the field‐assisted transfer of photoinduced charges from the channel to the polymer electret and localize these charges to improve memory stability. Therefore, phototransistor memory devices comprising 4) inactive polymer or 5) blocked floating gate electrets show improved memory stabilities with the assistance of gate bias to facilitate charge tunneling through the insulated barrier. To ensure the memory function without mutual interference, gate bias in device programming and reading was precluded by adopting 6) floating gate or 7) photoactive polymer electrets with a combined layer of photogate and insulated medium. This allows the insulated polymer to effectively protect the charges trapped in the floating gate and prevent charge leakage on both sides of the electret. Concisely, the design of photogate in electret is more competent than that of photogate in channel in nonvolatile memory application. Finally, phototransistor memory devices comprising 8) organic molecule electrets with isolated nanoarrays or layer structures have several advantages relative to polymer electrets, including ease of purification, well‐defined molecular structures, and ordered intermolecular packings. However, organic molecule electrets require elaborate nanostructure engineering to produce high performance without the need to apply gate bias to program or read the memory device.

## 4 Application of Phototransistors in Artificial Synapses and Photodetectors

In the previous sections, we focused on a literature review of phototransistor‐based memory devices. Here, we extend our horizon to review other applications of phototransistors, including artificial synapses (Section 4.1) and photodetectors (Section 4.2). Hysteresis to optical or electrical stimuli can induce memory effects in a phototransistor. Memory devices can be categorized as long‐term, short‐term, and sensor memory depending on their volatilities. For instance, an artificial synapse is regarded as STM device with medium volatility, whereas a photodetector should not show hysteresis to optical or electrical stimuli and will produce a completely volatile signal after removing external stimuli. Generally, phototransistors with strong hysteresis are used as memory devices; in contrast, phototransistors with negligible hysteresis are good photodetectors. Artificial synapses lie in between memory and sensors: depending on the operational history, such devices will have medium volatility and hysteresis to external stimuli.

### 4.1 Artificial Synapses

With the explosive growth in information generation, computation, and storage, computing systems following the von Neumann architecture (separated memory and central processing units) are beginning to face intrinsic limitations, making it increasingly important to reform the computing paradigm.^[^ ^121^ ^]^ In the previous sections, we introduced photoassisted information storage with phototransistor memory; here, we review recent advances in information computation using photosynaptic transistors.

The human brain contains ≈10^11^ neurons and ≈10^15^ synapses and possess the advantages of high parallelism and efficiency, fault tolerance, and low energy consumptions of only 1–100 fJ per synaptic event. It is particularly effective at handling the complex behaviors involved in cognitive learning, image recognition, and understanding language.^[^ ^122^ ^]^ It is therefore unsurprising that neuromorphic computing has attracted considerable attention in recent years. Among all the senses used for learning, more than 70% of information is derived from visual perception. Furthermore, synapses triggered by light pulses have unique advantages in terms of high bandwidth and interference immunity.^[^ ^123^ ^]^ Therefore, the development of photonic neuromorphic synapses represents a potential avenue to breaking the von Neumann bottleneck in photocommunication. The basic structure of a photosynaptic transistor is similar to that of a biological synapse, with source/drain electrodes corresponding to pre‐/postsynapses and light pulses corresponding to the action potentials applied presynapse. The carriers within the channel between the source/drain electrodes in a photosynaptic transistor are similar to the neurotransmitters within a synapse. Several significant parameters can be used as indicators to quantify synaptic efficacy. For example, the excitatory postsynaptic current (EPSC) or inhibitory postsynaptic current (IPSC) produced by light pulses in inducing an increase (carrier capture) or decrease (carrier release) in current primarily determines the degree of neuron connection (synaptic strength/weight). Synaptic plasticity relates to the ability of a synapse to precisely adjust its activity according to operational parameters including stimulus intensity and duration. Synaptic plasticity can be further divided into short‐term plasticity (STP) and long‐term plasticity (LTP) in accordance with STM and long‐term memory (LTM), respectively, which serve as the basis of learning and memory information processing in the human brain. Paired‐pulse facilitation (PPF) and paired‐pulse depression (PPD), referring to phenomena in which there is an increase (or decrease) in the ratio of postsynaptic current amplitude between two consecutive spikes, are direct indications of STP. In addition, the PPF index relies on a time interval that follows a biexponential function and plays an important role in identifying and decoding information. As mentioned previously, these parameters can be regulated by the intensity, wavelength, pulse number, frequency, duration of illumination, and gate/drain voltages applied to a photosynaptic transistor. In addition to STP, LTP is responsible for learning and memory retaining that last from hours to a whole life in the human brain, and LTP can be enabled by rehearsal learning process. In artificial synapses, LTP refers to the longstanding decay of EPSCs and thus can be modulated by increasing the synaptic weight, such as altering the number of pulses, pulse width, and interval between the pulse to achieve spike number‐dependent plasticity (SNDP), spike time‐dependent plasticity (STDP), and spike rate‐dependent plasticity (SRDP). Therefore, these functionalities enable LTP of artificial synapses and are of great importance to emulating LTM in human brain. In addition to the operation of artificial synapses, the most important artificial synapse parameter is energy consumption, defined as *I*_peak_ × *t* × *V*, where *I*_peak_, *t*, and *V* are the peak values of the EPSC (or IPSC), pulse duration time, and pulse voltage, respectively. In this section, we introduce photosynaptic devices with the following constituent materials: PVSK NCs, photoactive molecules, organic semiconductors, inorganic semiconductors owing to their respectable photoresponsivity and carrier mobility, and other material developments including metal NPs, ferroelectric gate, and gel electrolyte can be seen in other review literatures.^[^ ^121^, ^122^, ^123^ ^]^

**Figure 8:**
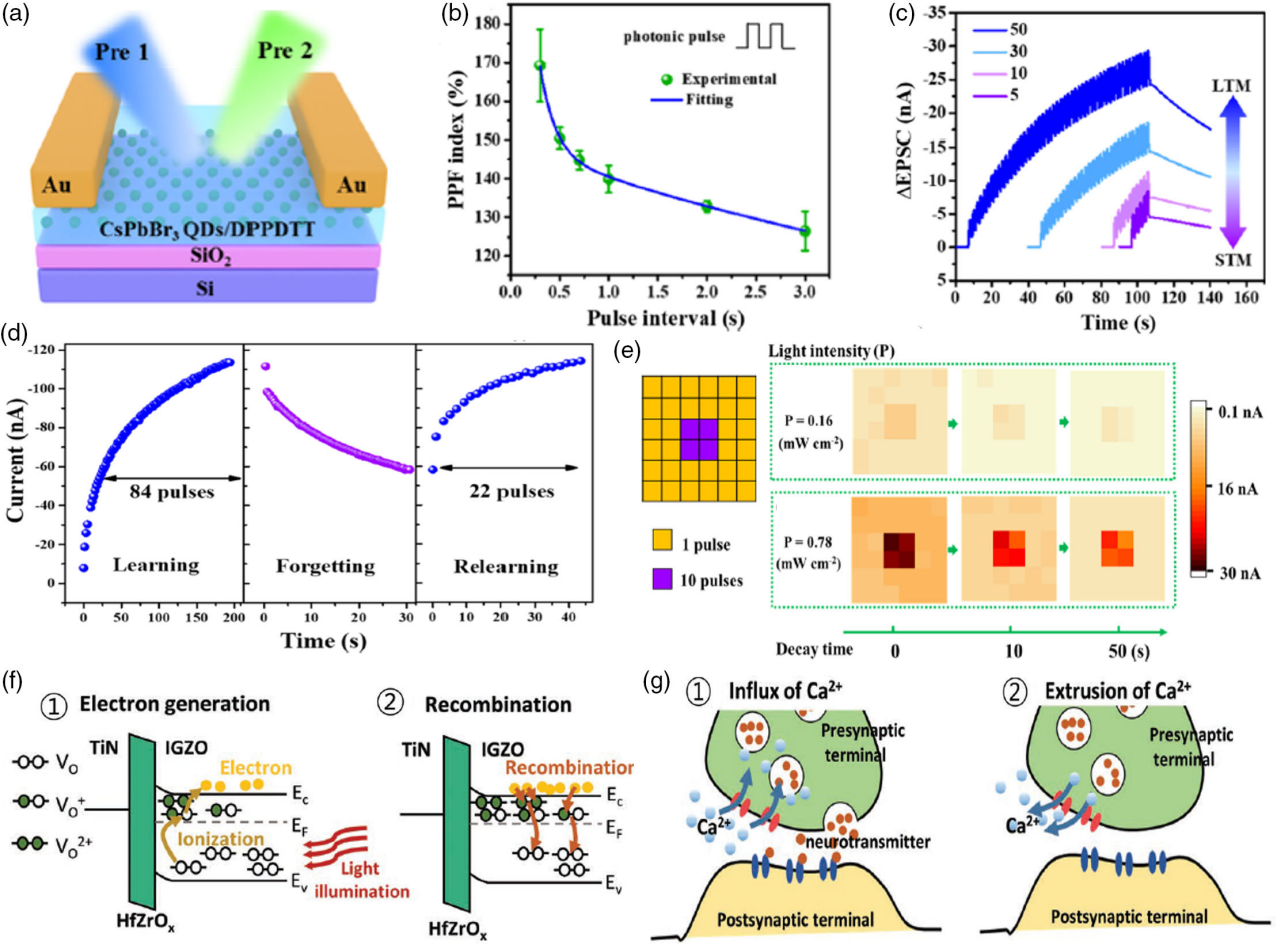
Photosynaptic transistor with channel‐containing photogates. a) Device architecture, b) correlation of the PPF index to the pulse interval, c) volatility transitions from STM to LTM by varying the pulse number, d) learning experience of the synaptic device including learning, forgetting, and relearning processes, and e) simulation of the neuromorphic visual system of DPPDTT/CsPbBr_3_ QD photosynaptic transistor. Reproduced with permission.^[^ ^126^ ^]^ Copyright 2020, American Chemical Society. f) Working mechanism of the photosynaptic transistor with metal oxide materials including the ionization and recombination processes of oxygen vacancies. g) Emulation of the photosynaptic transistor to the synaptic transmission in biological synapses, and the synaptic influx and extrusion processes of Ca^2+^. Reproduced with permission.^[^ ^132^ ^]^ Copyright 2020, Wiley‐VCH.

As a photosynaptic application of PVSK NCs, Wang et al. assessed the photonic potentiation and electrical habituation of a photosynaptic transistor based on a flash memory structure with a CsPbBr_3_ QD‐based blocked floating gate, a PMMA blocking layer, and a pentacene semiconducting channel. Their device could emulate synaptic functions such as STP, LTP, PPF, PPD, and SRDP at wavelengths of 365, 450, 520, and 660 nm. Photogenerated electrons and holes were trapped and released in the CsPbBr_3_ QDs. Under 1 s of illumination at intensities of 0.07, 0.13, and 0.15 mW cm^−2^, the EPSC gradually increased from 2.8 to 3.3 and finally to 4.6 nA, respectively, at *V*_DS_ = 0.2 V, enabling a transformation in memory volatility from STM to LTM synaptic plasticity. Evaluation of the PPF index at different light intensities and wavelengths revealed that an acceptable characteristic PPF ratio of 130% could be achieved at a wavelength of 365 nm, *V*_DS_ = 0.2 V, and intensity of 0.15 mW cm^−2^ with a preliminary energy consumption of 1.4 × 10^−9^ J event^−1^.^[^ ^124^ ^]^ In addition to simulating the behavior of photonic synapses, light pulses can play an auxiliary role in the functioning of electrical synaptic devices. Ham et al. fabricated a vertical, two‐terminal, bipolar artificial synapse containing CH_3_NH_3_PbI_3_ PVSK NCs that could mimic dopamine‐facilitated STP, LTP, and LTD synaptic activity under both electrical pulses and light stimulation. As the grain boundaries of the PVSK were sensitive to light irradiation and further weakened the conducting energy of the ions, illuminating the device inherently accelerated the migration of iodine vacancies to reduce the threshold of the LTP with very low programming input (0.1 V). A comparison of three conditions—1) electrical pulse‐only operation (pulse amplitude = 0.15 V, pulse width = 0.4 s); 2) light illumination‐only operation (intensity = 8.2 mW cm^−2^ for 0.4 s); and 3) combined electrical pulse and light illumination—revealed that light‐assisted electrical programming (3) produced the largest current at 3.2 × 10^−10^ A. Furthermore, they were able to attain a preliminary pattern recognition accuracy of 82% with a low power consumption of 4.8 nW.^[^ ^125^ ^]^ Photosynaptic transistors based on bilayer electret‐and‐channel structures generally require multiple processing steps and increase the fabrication cost and complexity, and the incorporation of high‐mobility, air‐stable semiconductors with photogates into such devices can simplify the fabrication procedure and achieve a faster photoresponse. As an example of this, Hao et al. implemented a configuration of high‐photosensitivity CsPbBr_3_ QDs/high‐mobility poly(diketopyrrolopyrrole–thienothiophene) (PDPPTT) as photonic synapses to mimic synaptic properties such as PPF, EPSC, transition of STM to LTM, and learning experience.^[^ ^126^ ^]^ The device architecture and correlation of PPF to pulse intervals under illumination at 450 nm and 0.26 mW cm^−2^ are shown in **Figure** **8**a,b. The photosynaptic assembly had a high PPF index of 170% at *V*_DS_ = −0.2 V and a time interval of 300 ms. Notably, by regulating the pulse number (5–50 pulses) and width (10–200 s) at 1 Hz, a clear STM–LTM transition process could be achieved, as shown in Figure 8c, as well as a learning–forgetting–relearning phenomenon (Figure 8d). Furthermore, the photosynaptic transistor could be programmed using 450 and 500 nm light to achieve basic AND/OR logic and neuromorphic visual functionality (Figure 8e). Impressively, the PDPPTT/CsPbBr_3_ QD photosynaptic transistor achieved a low energy consumption of 5 × 10^−16^ per spike at *V*_DS_ = −0.0005 V.

Very recently, Chen et al. used FA_*x*_Cs_1−*x*_PbBr_3_ (*x* = 0, 0.5, and 1)‐based PVSK QDs in blended FA‐(FAPbBr_3_), mixed FA/Cs‐(FA_0.5_Cs_0.5_PbBr_3_), and Cs‐(CsPbBr_3_) QD‐and‐P3HT‐based photomemory to imitate the optical potentiation and electric depression of photosynaptic transistors, PPF, EPSC, LTP, and STM–LTM transitions, and multiple spike‐dependent plasticity synaptic behaviors. The P3HT and QDs served as charge‐transporting and trapping elements among the composites, effectively separating the charge pairs to reduce the illumination time. Using FA‐QDs, which have the lowest valence band and the longest exciton lifetime, shortened the light‐recordable time to 1 s and achieved acceptable relaxation times of *τ*_1_ = 1367 and *τ*_2_ = 15.8 ms, demonstrating their stable and fast photoresponses. A photosynaptic transistor fabricated from FA‐QDs/P3HT was able to carry out both optical potentiation (10 optical pulses, 6 mW cm^−2^, 1 s duration, 10 s interval) and electric depression (−40 V, 1 s duration, 20 s interval). P3HT/PVSK QD composite films of P3HT and PVSK QDs were able to act as charge‐transporting materials with improved photoinduced charge‐trapping sites. As an example of the usefulness of enhanced charge trapping, a mixed heterojunction layer configuration can simplify device structure and increase the contact area between the P3HT and PVSK relative to a bilayer configuration comprising a floating gate or polymer electret under a semiconducting channel, allowing for the effective separation of electrons and holes within an extremely short time. Notably, the FA‐QDs/P3HT photosynaptic transistor exhibited an ultralow energy consumption of 3 × 10^−17 ^J with EPSC = 1.2×10^−12^ A collected at a *V*_DS_ of −5 × 10^−4 ^V and a spike pulse of 5 × 10^−2^ s under illumination at 450 nm; this represents state‐of‐the‐art artificial synapse behavior with the lowest energy consumption reported to date.^[^ ^127^ ^]^ Subsequently, Ercan et al. demonstrated the efficacy of self‐assembled nanostructures in photosynaptic transistors composed of CsPbBr_3_ QDs and P3HT.^[^ ^128^ ^]^ By introducing marginal solvent of acetonitrile, ultrasonication, and UV treatment to the blending solution of QDs and P3HT, QDs formed aligned coaggregates along with the nanofibrillar P3HT, thereby achieving a decent synaptic performance of a low energy consumption of 1.8 × 10^−16 ^J with EPSC = 3.6 × 10^−11^ A collected at *V*_DS_ of −0.001 V and a spike pulse of 5 × 10^−2^ s under illumination at 450 nm to the previous result. It is worth noting that CsPbBr_3_ QD presents lower charge transfer efficiency to P3HT than FAPbBr_3_ QDs based on the time‐resolved photoluminescent characteristics,^[^ ^127^ ^]^ and therefore, further advancement is expected to be achieved by combining high‐performance QDs and nanostructured engineering to the semiconductors and photogates.

Although PVSK materials provide a wide range of advantages as powerful photogates in phototransistors, they are organically detrimental and have the potential to cause environmental pollution. Following an enhanced awareness of the need for environmental protection, phototransistors are now combined with biomaterials to improve their biocompatibility and environmental sustainability. For example, Lv et al. fabricated a floating gate using carbon dots and silk protein (CDs/silk) as a charge‐trapping medium to enable dual‐mode modulation (volatile/nonvolatile) of a photosynaptic transistor with five well‐defined resistance states.^[^ ^129^ ^]^ Owing to its high transparency and water solubility, silk protein was used as the biopolymer matrix to improve the stability of the CDs. In nonvolatile mode, their device achieved acceptable storage capability, programming reproducibility, and long‐term stability over 7 × 10^6^ s. By regulating the input optical pulses (temporal and permanent), the channel current could be made analogous to STP and LTP in volatile mode. By appropriately combining optical and electrical stimuli, the EPSC feature could be triggered to obtain a PPF ratio of up to 124% at a wavelength of 365 nm, intensity of 0.15 mW cm^−2^, and *V*_DS_ = −1 V. Conversely, a PPD behavior of −168% could be achieved by applying a suitable gate bias to the phototransistor. Furthermore, they demonstrated a flexible phototransistor using Al_2_O_3_ as a blocking dielectric layer with a low operation voltage of −5 V. Finally, they developed an artificial neural network comprising a single perception layer that achieved good accuracy of 73% in pattern recognition.

Using a suitable structural design, multifunction phototransistors that can, for instance, engage in both synaptic and memory behavior can be implemented on a single device. For example, Yang et al. fabricated multifunctional photoresponsive transistor memory based on blends of chlorophyll biomaterial and a PDPP2T semiconductor. Their devices could achieve photodetector or photosynaptic transistor functionalities by switching the negative or positive gate voltages.^[^ ^130^ ^]^ Several important synaptic functions, including EPSC, PPF, STM, LTM, learning and forgetting, and image‐processing functions, were also demonstrated. Fitting the PPF index under illumination at 430 nm (intensity = 1 mW cm^−2^, *V*_DS_ = −1 V) by a biexponential function resulted in time constants of *τ*_1_ = 82 and *τ*_2_ = 1479 ms, indicating a time scale appropriate for a biological synapse. Notably, the device could be operated at *V*_DS_ = −10^−5^ V with spike widths of up to 50 ms, corresponding to a calculated energy consumption per spike of 2.5 × 10^−16^ J. The pattern recognition capability of the photosynaptic transistor was further demonstrated by applying it in a convolutional neural network. In addition to the architecture of channel with photogate, Wang et al. subsequently fabricated an ultrasensitive photosynaptic transistor using bilayered structure comprising DNTT and hydoxy/nitophenyl‐porphyrin (TPP) that could detect weak light signals with an intensity of 1 μW cm^−2^ or transient light stimuli as short as 0.05 s.^[^ ^131^ ^]^ PPF and EPSC synaptic behaviors and the transition of synaptic function between STP and LTP could be triggered using 450 nm light, with a PPF index of 126% obtained under light illumination for 0.5 s at an intensity of 160 μW cm^−2^. Notably, the synaptic device could operate at an ultralow voltage of −70 μV, obtaining a low energy consumption of 1.4 × 10^−15^ J under a light intensity of 12 μW cm^−2^.

In addition to organic photogates and semiconductors, inorganic semiconductors are promising materials for use in high‐performance photosynaptic transistors. Their synaptic properties are primarily derived from the persistent photoconductivity (PPC) and related relaxation phenomena of oxide semiconductors. The dynamics of PPC are similar to those of Ca^2+^ in biological synapses, and most of the generation and recombination mechanisms, in which oxygen vacancies are ionized under light stimulation and become positively charged, are caused by the slow deionization process that occurs after turning off light stimulation. Kim et al. controlled the PPC and relaxation characteristics of an oxide semiconductor of indium gallium zinc oxide (IGZO) and a polarizable ferroelectric layer (HfZrO_*x*_) to construct an oxide‐based layer TiN/HfZrO_*x*_/IGZO/Al structure.^[^ ^132^ ^]^ The working mechanism of their photosynaptic transistor and its emulation of synaptic transmission in a biological synapse are shown in Figure 8f,g. In their device, optical stimulus increased the conductance of IGZO and the polarization of HfZrO_*x*_ successfully induced charge accumulation and depletion without an external power supply. Further evaluation of STP, PPF, and LTP synaptic behaviors revealed a high conductance amplification of 280% and EPSC retention of 28% in a device with downward polarization. This device outperformed one with upward polarization, which had a conductance amplification of only 200% and an EPSC retention of only 1% after 250 s.

### 4.2 Photodetectors

Phototransistors have been consistently spotlighted as promising candidates for use in photodetectors owing to their light weight and compatibility with circuit integration. In addition, they achieve highly tunable device performance by applying a gate voltage to induce gate effects to allow the channel conductivity to be configured by both gate voltage and light.^[^ ^133^ ^]^ Compared with other photodetectors, device configurations such as photodiodes and photoconductors, phototransistors have more concentrated carrier concentrations in channel, which are conducive to achieving high photosensitivity. Notably, phototransistor‐based near‐infrared detectors can be used in a wide range of emerging applications including noninvasive bioimaging, wearable health monitoring, and biometric authentication owing to their mechanical flexibility, affordability, and solution processability.^[^ ^134^, ^135^, ^136^ ^]^

The material systems used in phototransistor‐based photodetectors comprise 1) semiconductors and 2) photoabsorbers, which determine, respectively, the charge transport polarity and photoresponse of the phototransistor. It is possible to combine these two functionalities into a single semiconducting polymer material or molecule for use in a photodetector. Park et al. reported a near‐infrared organic phototransistor with a p‐channel based on poly(diketopyrrolopyrrole‐benzothiadiazole) (PDPPBT) with a narrow bandgap.^[^ ^137^ ^]^ In thin films, close contact between DPP units can cause strong dipole interactions between carbonyl groups and nearby nitrogen atoms. Therefore, evenly distributed charge transfer complexes were formed in the crystalline domain of the channel layer to promote the photoresponse. The device provided preliminary photoresponsivities of 0.014–0.015 A W^−1^ to 830–935 nm light with an intensity of 0.34 mW cm^−2^.

To achieve extended compatibility in circuit designs using organic phototransistors, it is necessary to secure both the p‐ and n‐channels in the phototransistor. However, most organic transistors use p‐type‐conjugated polymers, forcing the resulting device to run in p‐channel mode. There is, in contrast, a very limited range of n‐type conjugated polymers available for use in n‐channels. Recently, Lee et al. reported a stable n‐type phototransistor comprising poly(dithieno‐indacenedithiophene‐naphthalene diimide) that could absorb blue, red, and near‐IR light.^[^ ^138^ ^]^ The device demonstrated preliminary photoresponsivities of 0.015 and 0.018 A W^−1^ to 434 and 754 nm light, respectively, with an intensity of 0.1 mW cm^−2^. In addition to direct application to semiconductors as channels and photoabsorbers, photoabsorbers can be blended with semiconductors to form bulk heterojunctions or stacked with semiconductors to form heterostructure systems. To achieve this, effective generation and dissociation of charges must be possible, presumably aided by the application of gate voltage, and interfaces such as bulk heterojunctions or bilayers are required for effective dissociation within the organic semiconductor. Furthermore, balanced charge transport pathways for both electrons and holes must be possible within the channel.

A simple approach to fabricating photodetectors is the blending of semiconductors and photoabsorbers. Herrera et al. reported an n‐type phototransistor comprising blends of P3HT and PCBM.^[^ ^139^ ^]^ Their results indicated that a PCBM channel with 1 wt% P3HT was sufficient to induce photoconductivity with unipolar electron transport. However, they found that photoinduced charge excitation and carrier transport within the same channels increased the recombination losses of photoexcited charge carriers and electrical noise, thereby reducing the detectable amplitude range of the optical signal. A heterostructure system, in contrast, can maintain and promote the discrete characteristics, for example, high mobility, strong optical absorption, and strong emission, of two different materials. Chu et al. reported a phototransistor with a DNTT channel and a polylactic acid (PLA) polar dielectric.^[^ ^140^ ^]^ Their device achieved enhanced photosensitivity, which they explained based on a multiple trap‐and‐release model in which the polar groups in the PLA film can induce high‐density charge traps at different energy levels at the organic semiconductor/dielectric interface, where the majority of charge carriers are concentrated. This charge trapping effect in PLA can reduce the carrier transportation rate by temporarily trapping carriers, resulting in an ultralow dark current. In contrast, the DNTT channel provides fast transportation paths for photogenerated charges, while maintaining a low dark current and high current contrast. Their device demonstrated a high current contrast of >3,000−450 nm light at an intensity of 100 mW cm^−2^.

**Figure 9:**
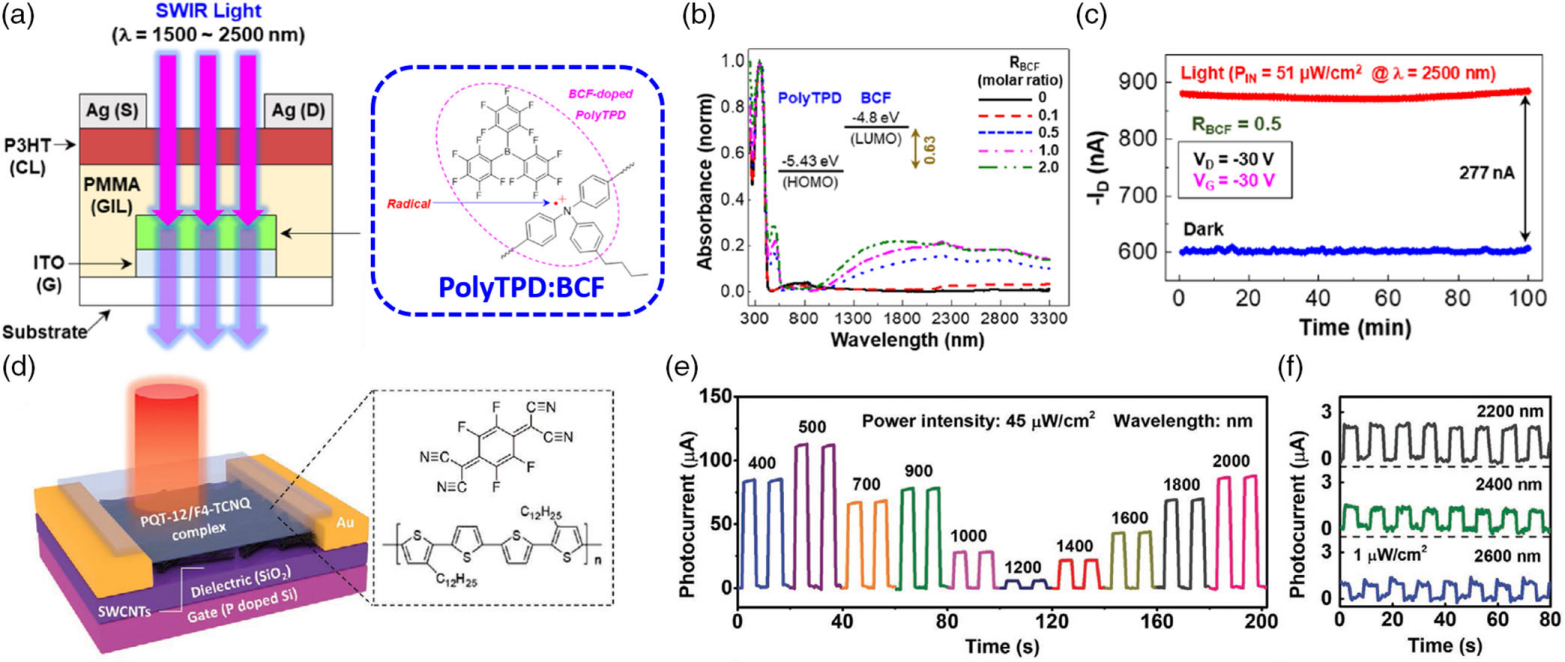
Photodetectors with a blocked photoabsorber design. a) Device architecture and chemical structures of polyTPD and BCF. b) Optical absorption spectra of the polyTPD:BCF films with various BCF contents (inset: offset between the HOMO energy level of polyTPD and the LUMO energy level of BCF). c) Current state in the dark or irradiated by 2500 nm light of the phototransistor comprising a photoabsorber of polyTPD:BCF, a blocking layer of PMMA, and a channel of P3HT. Reproduced with permission.^[^ ^141^ ^]^ Copyright 2021, American Chemical Society. Photodetector with charge transfer complex as a photoabsorber. d) Device architecture and chemical structures of PQT and F4‐TCNQ. Photoresponses of the photodetector to different wavelengths of light at e) 45 μW cm^−2^ and at f) 1 μW cm^−2^. Reproduced with permission.^[^ ^145^ ^]^ Copyright 2021, Wiley‐VCH.

Polymer dielectric insulators can be good matrices for accommodating photoabsorbers; this feature has led to a number of studies on the combination of insulated polymers and photoabsorbers into blocked or floating designs. A blocked design is constructed by installing a polymer insulator between a semiconducting channel and photoabsorber layer, whereas a floating design is achieved by mixing a polymer insulator with a photoabsorber and placing the blend above or below the channel to absorb light. For example, Lee et al. reported a photoabsorber comprising poly(triphenylamine) and tris(pentafluorophenyl)borane (BCF) in which the photoabsorber layer was blocked by PMMA.^[^ ^141^ ^]^ The chemical structures of the constituent materials and their device architecture are shown in **Figure** **9**a. BCF doping was found to create unpaired electrons (radicals) in the HOMO levels of poly(triphenylamine), producing broad absorption covering the full range of the short‐wave infrared (Figure 9b). The photoresponses of their photodetector device in the dark and under irradiation with 2500 nm light are shown in Figure 9c. The resulting device achieved good photoresponsivities to 0.58, 0.70, 0.83, 1500, 2000, and 2500 nm light with intensities of 50–70 μW cm^−2^. Lim et al. reported a phototransistor based on a floating design in which the photoabsorber was blended with PS and formed a poly(diketopyrrolopyrole–thienothiophene) (PDPPTT) semiconductor.^[^ ^142^ ^]^ The photoabsorber molecule, which had a twisted conjugation comprising a donor–acceptor–donor design of triphenylamine−difluorobenzothiadiazole−pyrene, had a broadband detection range spanning 250−700 nm. Triphenylamine and pyrene donors were used as hole transport pathways, with the difluorobenzothiadiazole acceptor serving as the electron trap and tetracyanoethylene between the donor and acceptor moieties was used to prevent charge leakage. The resulting device had a preliminary photoresponsivity of 0.09 A W^−1^ to 400 nm light with an intensity of 1.1 mW cm^−2^.

A polymer insulator can potentially hinder charge tunneling and exciton dissociation to produce a modest photoresponse. Recent studies have reported high‐performance phototransistors that achieve heterojunction bilayer structures without the use of polymer insulators. Chen et al. reported a phototransistor with a CsPbBr_3_ QD photoabsorber layer and a DNTT channel with a high photoresponsivity of 1.7 × 10^4 ^A W^−1^ and a photodetectivity of 2 × 10^14^ Jones to 460 nm light with an intensity of 1 mW cm^−2^.^[^ ^143^ ^]^ The good performance of their design can be attributed to fast interfacial charge dissociation. During illumination, a large number of charges are generated in both the channel and photoabsorber layers. These charge pairs quickly dissociate near the interfaces owing to potential bias, and the holes are transported from the QDs to the DNTT and electrodes, which is favorable to the separation of the holes and electrons and reduces their recombination. Li et al. reported an air‐stable, n‐type, near‐IR sensor based on a poly{5,5’‐bis[3,5‐bis(thienyl)phenyl]‐2,2’‐bithiophene‐3‐ethylesterthiophene} (PTPBT‐ET) photoabsorber layer and an In_2_O_3_ channel with a good photoresponsivity of 200 A W^−1^ and a photodetectivity of 2 × 10^13^ Jones to 850 nm light with an intensity of 0.2 mW cm^−2^.^[^ ^144^ ^]^ The good performance of this design can be attributed to the high electron mobility of In_2_O_3_ and the high‐NIR absorbance of PTPBT‐ET. It is understood that the formation of charge transfer complexes can effectively broaden optical absorption and improve the photoresponse of a phototransistor. For example, Yang et al. reported an IR sensor comprising a charge transfer complex of poly(3,3’’’‐dialkylquaterthiophene) (PQT‐12) and 2,3,5,6‐tetrafluoro‐7,7,8,8‐tetracyanoquinodimethane (F_4_‐TCNQ) in which single‐walled carbon nanotubes (SWCNTs) were used as p‐type channels.^[^ ^145^ ^]^ The device architecture and chemical structures of the constituent materials are shown in Figure 9d. Benefiting from the ultralow electronic transition energy of the charge transfer complex between PQT and F_4_‐TCNQ, the phototransistor achieved broadband photodetection spanning the range of 400−2600 nm, and the resulting device exhibited an ultrahigh photoresponsivity of 2.8 × 10^6 ^A W^−1^ and a photodetectivity of 3 × 10^14^ Jones under illumination by 2000 nm light with an intensity of 45 μW cm^−2^ (Figure 9e,f). Notably, their photodetector with charge transfer complexes could respond to blackbody irradiation at 600 and 700 °C, a practical temperature region for industry, scientific research, and national defense purposes that is rarely reported for organic phototransistors. Finally, with the growing demand for UV−vis−IR detectors, myriads of materials systems will be consistently explored with the goal of achieving systems with broadband, low‐intensity, and fast phototransistor detection.

## 5 Conclusion and Future Outlook

In this review, we discussed the recent progress in phototransistors and their diverse applications, including nonvolatile memory, artificial synapses, and photodetectors. After introducing the basic design concepts, requirements, and architectures of phototransistor memory, we systematically categorized nonvolatile memory device architectures, including channel‐only, channel‐with‐photogate, and photochromatic‐channel devices, as well as devices incorporating floating gate, photoactive polymer, and organic molecule‐based electrets. Based on what we have learnt from this literature review, we can draw some key points. First, a phototransistor memory device without an electret is favorable for fast response applications but usually requires gate bias to program or even read the device, with the resulting relatively poor memory retention presenting a significant problem. However, this design is revealed to be viable to achieve high‐performance photodetectors and artificial synapses, having higher volatilities than memory devices. Next, by introducing a polymer electret below the channel, it is possible to achieve the field‐assisted transfer of photoinduced charges from the channel to the polymer electret and localize these charges to improve memory stability. Based on this, designs with floating gate and photoactive polymer electrets are potential candidates for use in high‐performance memory devices. Floating gate electrets can be prepared via a one‐step spin‐coating technique, avoiding the need for the multiple process steps applied in constructing the blocked floating gate architecture. This allows the insulated polymer to effectively protect the charges trapped in the floating gate and prevent charge leakage on both sides of the electret. The proliferation of floating gate electret designs based on polymer dots, inorganic QDs, and PVSK NCs should further advance the development of high‐performance phototransistor memory. Photoactive polymer electrets have bulk homogeneity, acceptable mechanical tolerances, and more diversified structural design potential than hybrid composites. Among these, donor–acceptor polymers and conjugated BCPs have been shown to be capable of achieving high performance in phototransistor memory. Trimming the electron‐donating/accepting groups of donor–acceptor polymers modulates their energy levels and optical absorption and regulates the polarity and storage of the trapped charges within them. In contrast, fine tuning the compositions of conjugated BCPs can modulate their photoresponse and charge retention capabilities. Organic molecules have several advantages relative to polymer electrets, including ease of purification, batch‐to‐batch reproducibility, well‐defined molecular structures, and ordered intermolecular packings. Organic molecules can also be engineered with isolated nanostructures (such as nanocolumns) or layered structures to achieve high performance without the need to apply gate bias to program or read the memory device.

Because the absorption band, energy‐level alignment, and morphology of the interface of the channel and memory layers are all vital to memory behavior; a well‐chosen combination of the respective materials plays an important role in memory performance. The following issues must be addressed and requirements met to enhance memory performance and understand the memory mechanism. 1) Reliable multiresponsiveness can be achieved utilizing photochromic molecules or exploiting the ferroelectricity of a gate insulator in a transistor memory. To this end, ferroelectric phototransistor memory is promising in terms of achieving multibit data storage within unit cells. 2) The adoption of a vertical structure can shorten the length of the charge transfer channel from tens of micrometers to the order of nanometers, effectively reducing the trapped charge leakage and eventually allowing for multilevel storage with high discrepancies and long‐term retention characteristics.^[^ ^57^, ^58^, ^59^, ^60^, ^61^, ^62^ ^]^ 3) Most organic molecules cannot absorb in the IR band, which is critical for data encryption technology. Using upconversion NCs, high‐energy photons can be emitted at the expense of two or more low‐energy photons. Eventually, the high‐energy emission from upconversion materials is reabsorbed by the channel to alter the charge‐trapping efficiency and increase the data storage level of the memory device. In addition, charge transfer complexes with broadband optical absorption could be further investigated to fulfill the requirements of IR‐programming memory devices. 4) Polymer‐based insulated dielectrics without current leakage should be developed to enable flexible or even stretchable memory devices that can accommodate the development of wearable electronics. 5) Considering the extended compatibilities required in circuit designs, it is necessary to secure both p‐ and n‐channels in organic phototransistors. However, most conjugated polymers used in these devices are p‐type and therefore limit device functioning to the p‐channel mode, making it important to accelerate the development of n‐type phototransistors for use in nonvolatile memory, artificial synapses, and photodetectors. 6) The trapping mechanism in a memory device is believed to involve the pairing of two mobile holes to form a nonconducting bipolaron. As this differs from the most common charge‐trapping mechanism, a more specific technique such as charge modulation spectroscopy should be applied to investigate the trapping mechanism in phototransistors.^[^ ^146^, ^147^, ^148^, ^149^ ^]^

In addition to their uses in conventional memory devices, it will be important to integrate the photodetecting and photoprogramming properties of photodetectors into brand new applications such as image sensing systems, data encryption, or even robot visual perception as part of artificial intelligence systems. Such applications could help trigger even more possibilities for the combination of smart electronics that will improve life in the future. The development of photodetector‐based artificial visual systems could complement recent developments in the field of machine learning to enable image recognition. Another growing area of research is the development of biomimetics using optically driven devices that mimic neural systems. In such systems, light pulses and electrical stresses could be utilized to stimulate artificial synapses that can imitate human learning and forgetting behaviors. Research in this area could guide in the future development of phototransistors with better performance as well as novel potential applications. An increasing number of researchers from different backgrounds are now working on the development of phototransistors, and it is expected that their efforts will lead to devices that can provide advanced contributions to sensing systems in the near future.

## Conflict of Interest

The authors declare no conflict of interest.

## References

[1] F. Zhou , Z. Zhou , J. Chen , T. H. Choy , J. Wang , N. Zhang , Z. Lin , S. Yu , J. Kang , H.-S. P. Wong , Nat. Nanotechnol. 2019, 14, 776. 31308498 10.1038/s41565-019-0501-3

[2] L. Mennel , J. Symonowicz , S. Wachter , D. K. Polyushkin , A. J. Molina-Mendoza , T. Mueller , Nature 2020, 579, 62. 32132692 10.1038/s41586-020-2038-x

[3] M. D. Tran , H. Kim , J. S. Kim , M. H. Doan , T. K. Chau , Q. A. Vu , J. H. Kim , Y. H. Lee , Adv. Mater. 2019, 31, 1807075. 10.1002/adma.201807075 30589128

[4] Q. Xia , M. D. Pickett , J. J. Yang , X. Li , W. Wu , G. Medeiros-Ribeiro , R. S. Williams , Adv. Funct. Mater. 2011, 21, 2660.

[5] S. Hong , S. H. Choi , J. Park , H. Yoo , J. Y. Oh , E. Hwang , D. H. Yoon , S. Kim , ACS Nano 2020, 14, 9796. 32628447 10.1021/acsnano.0c01689

[6] C. Choi , J. Leem , M. S. Kim , A. Taqieddin , C. Cho , K. W. Cho , G. J. Lee , H. Seung , H. J. Bae , Y. M. Song , Nat. Commun. 2020, 11, 1. 33230113 10.1038/s41467-020-19806-6 PMC7683533

[7] S. M. Kwon , S. W. Cho , M. Kim , J. S. Heo , Y. H. Kim , S. K. Park , Adv. Mater. 2019, 31, 1906433. 10.1002/adma.201906433 31725185

[8] H. Han , H. Yu , H. Wei , J. Gong , W. Xu , Small 2019, 15, 1900695. 10.1002/smll.201900695 30972944

[9] H. Wu , Z. Kang , Z. Zhang , H. Si , S. Zhang , Z. Zhang , Q. Liao , Y. Zhang , Small Methods 2019, 3, 1900117.

[10] C. Xie , C. K. Liu , H. L. Loi , F. Yan , Adv. Funct. Mater. 2020, 30, 1903907.

[11] Y. Hou , L. Wang , X. Zou , D. Wan , C. Liu , G. Li , X. Liu , Y. Liu , C. Jiang , J. C. Ho , Small 2020, 16, 1905609. 10.1002/smll.201905609 31899596

[12] P. Balakrishna Pillai , M. M. De Souza , ACS Appl. Mater. Interfaces 2017, 9, 1609. 27990819 10.1021/acsami.6b13746

[13] Y. Nishitani , Y. Kaneko , M. Ueda , T. Morie , E. Fujii , J. Appl. Phys. 2012, 111, 124108.

[14] N. Huo , G. Konstantatos , Adv. Mater. 2018, 30, 1801164. 10.1002/adma.201801164 30066409

[15] F. Yan , Z. Wei , X. Wei , Q. Lv , W. Zhu , K. Wang , Small Methods 2018, 2, 1700349.

[16] Y. S. Rim , Y. Yang , S. H. Bae , H. Chen , C. Li , M. S. Goorsky , Y. Yang , Adv. Mater. 2015, 27, 6885. 26423662 10.1002/adma.201502996

[17] F. Yang , S. Cheng , X. Zhang , X. Ren , R. Li , H. Dong , W. Hu , Adv. Mater. 2018, 30, 1702415. 10.1002/adma.201702415 29024065

[18] P. Gu , Y. Yao , L. Feng , S. Niu , H. Dong , Polym. Chem. 2015, 6, 7933.

[19] C. Zou , Y. Xi , C. Y. Huang , E. G. Keeler , T. Feng , S. Zhu , L. D. Pozzo , L. Y. Lin , Adv. Opt. Mater. 2018, 6, 1800324.

[20] X. Chen , X. Liu , B. Wu , H. Nan , H. Guo , Z. Ni , F. Wang , X. Wang , Y. Shi , X. Wang , Nano Lett. 2017, 17, 6391. 28876943 10.1021/acs.nanolett.7b03263

[21] B. Zhao , Z. Gan , M. Johnson , E. Najafidehaghani , T. Rejek , A. George , R. H. Fink , A. Turchanin , M. Halik , Adv. Funct. Mater. 2021, 31, 2105444.

[22] C.-C. Shih , W.-Y. Lee , W.-C. Chen , Mater. Horiz. 2016, 3, 294.

[23] Z. Zhu , Y. Guo , Y. Liu , Mater. Chem. Front. 2020, 4, 2845.

[24] Y. Ni , Y. Wang , W. Xu , Small, 2021, 17, 1905332. 10.1002/smll.201905332 32243063

[25] X. Feng , X. Liu , K.-W. Ang , Nanophotonics 2020, 9, 1579.

[26] A. K. Tripathi , A. J. J. M. van Breemen , J. Shen , Q. Gao , M. G. Ivan , K. Reimann , E. R. Meinders , G. H. Gelinck , Adv. Mater. 2021, 23, 4146. 10.1002/adma.201101511 21818787

[27] H. Yoo , I. S. Lee , S. Jung , S. M. Rho , B. H. Kang , H. J. Kim , Adv. Mater. 2021, 33, 2006091. 10.1002/adma.202006091 34048086

[28] W. Li , F. Guo , H. Ling , H. Liu , M. Yi , P. Zhang , W. Wang , L. Xie , W. Huang , Small 2018, 14, 1701437. 10.1002/smll.201701437 29165914

[29] Y. Zhai , J.-Q. Yang , Y. Zhou , J.-Y. Mao , Y. Ren , V. A. L. Roy , S.-T. Han , Mater. Horiz. 2018, 5, 641.

[30] Y. Yu , Q. Ma , H. Ling , W. Li , R. Ju , L. Bian , N. Shi , Y. Qian , M. Yi , L. Xie , Adv. Funct. Mater. 2019, 29, 1904602.

[31] C. Liu , H. Chen , X. Hou , H. Zhang , J. Han , Y.-G. Jiang , X. Zeng , D. W. Zhang , P. Zhou , Nat. Nanotechnol. 2019, 14, 662. 31133664 10.1038/s41565-019-0462-6

[32] R. Han , Y. Xiang , P. Huang , Y. Shan , X. Liu , J. Kang , Adv. Intell. Syst. 2021, 3, 2000161.

[33] Q. Zhang , T. Jin , X. Ye , D. Geng , W. Chen , W. Hu , Adv. Funct. Mater. 2021, 31, 2106151.

[34] Z. Lv , Y. Zhou , S.-T. Han , V. Roy , Mater. Today 2018, 21, 537.

[35] Z. Lv , Y. Wang , J. Chen , J. Wang , Y. Zhou , S.-T. Han , Chem. Rev. 2020, 120, 3941. 32202419 10.1021/acs.chemrev.9b00730

[36] Y. R. Lee , T. Q. Trung , B.-U. Hwang , N.-E. Lee , Nat. Commun. 2020, 11, 2753. 32488078 10.1038/s41467-020-16606-w PMC7265430

[37] Y. Lee , H.-L. Park , Y. Kim , T.-W. Lee , Joule 2021, 5, 794.

[38] Y. Huang , E. Sutter , J. T. Sadowski , M. Cotlet , O. L. Monti , D. A. Racke , M. R. Neupane , D. Wickramaratne , R. K. Lake , B. A. Parkinson , ACS Nano 2014, 8, 10743. 25247490 10.1021/nn504481r

[39] M. M. Furchi , D. K. Polyushkin , A. Pospischil , T. Mueller , Nano Lett. 2014, 14, 6165. 25299515 10.1021/nl502339q

[40] H. Xu , J. Xing , J.-H. Lu , X. Han , D. Li , Z. Zhou , L.-H. Bao , H.-J. Gao , Y. Huang , Appl. Surf. Sci. 2019, 484, 39.

[41] W. Kim , A. Javey , O. Vermesh , Q. Wang , Y. Li , H. Dai , Nano Lett. 2003, 3, 197. 10.1021/nl034010k 36517998

[42] A. Salleo , R. A. Street , Int. J. Appl. Phys. 2003, 94, 471.

[43] S. Dutta , K. S. Narayan , Adv. Mater. 2004, 16, 2151.

[44] M. Ujimoto , W. Takashima , K. Kaneto , Thin Solid Films 2006, 499, 313.

[45] Y.-Y. Noh , J. Ghim , S.-J. Kang , K.-J. Baeg , D.-Y. Kim , K. Yase , Int. J. Appl. Phys. 2006, 100, 094501.

[46] M. Y. Cho , S. J. Kim , Y. D. Han , D. H. Park , K. H. Kim , D. H. Choi , J. Joo , Adv. Funct. Mater. 2008, 18, 2905.

[47] L. Zhen , L. Shang , M. Liu , D. Tu , Z. Ji , X. Liu , G. Liu , J. Liu , H. Wang , Appl. Phys. Lett. 2008, 93, 203302.

[48] M. Barra , F. Bloisi , A. Cassinese , F. V. Di Girolamo , L. Vicari , Int. J. Appl. Phys. 2009, 106, 126105.

[49] Z. Qi , X. Liao , J. Zheng , C.-A. Di , X. Gao , J. Wang , Appl. Phys. Lett. 2013, 103, 053301.

[50] A. Li , X. Wei , Y. He , C. He , M. U. Ali , H. Yang , O. Goto , H. Meng , Appl. Phys. Lett. 2018, 113, 103301.

[51] K. Pei , X. Ren , Z. Zhou , Z. Zhang , X. Ji , P. K. L. Chan , Adv. Mater. 2018, 30, 1706647. 10.1002/adma.201706647 29424125

[52] L. Zheng , J. Li , Y. Wang , X. Gao , K. Yuan , X. Yu , X. Ren , X. Zhang , W. Hu , Nanoscale 2019, 11, 7117. 30919870 10.1039/c9nr00578a

[53] C.-C. Chen , M.-Y. Chiu , J.-T. Sheu , K.-H. Wei , Appl. Phys. Lett. 2008, 92, 143105.

[54] M.-Y. Chiu , C.-C. Chen , J.-T. Sheu , K.-H. Wei , Org. Electron. 2009, 10, 769.

[55] Y. Zhou , S. T. Han , X. Chen , F. Wang , Y. B. Tang , V. A. Roy , Nat. Commun. 2014, 5, 4720. 25144762 10.1038/ncomms5720

[56] E. Ercan , Y. C. Lin , L. C. Hsu , C. K. Chen , W. C. Chen , Adv. Mater. Technol. 2021, 6, 2100080.

[57] H. Yang , Y. Liu , X. Wu , Y. Yan , X. Wang , S. Lan , G. Zhang , H. Chen , T. Guo , Adv. Electron. Mater. 2019, 5, 1900864.

[58] X. Wu , S. Lan , D. Hu , Q. Chen , E. Li , Y. Yan , H. Chen , T. Guo , J. Mater. Chem. C 2019, 7, 9229.

[59] T. Chen , X. Wang , D. Hao , S. Dai , Q. Ou , J. Zhang , J. Huang , Adv. Opt. Mater. 2021, 9, 2002030.

[60] C. Gao , H. Yang , E. Li , Y. Yan , L. He , H. Chen , Z. Lin , T. Guo , ACS Photonics 2021, 8, 3094.

[61] M. Sulaman , Y. Song , S. Yang , M. Li , M. I. Saleem , P. V. Chandraseakar , Y. Jiang , Y. Tang , B. Zou , Nanotechnology 2019, 31, 105203. 31751965 10.1088/1361-6528/ab5a26

[62] A. Subramanian , S. Hussain , N. Din , G. Abbas , A. Shuja , W. Lei , J. Chen , Q. Khan , K. Musselman , ACS Appl. Electron. Mater. 2020, 2, 3871.

[63] H. Yang , Y. Yan , X. Wu , Y. Liu , Q. Chen , G. Zhang , S. Chen , H. Chen , T. Guo , J. Mater. Chem. C 2020, 8, 2861.

[64] S. Lan , J. Zhong , E. Li , Y. Yan , X. Wu , Q. Chen , W. Lin , H. Chen , T. Guo , ACS Appl. Mater. Interfaces 2020, 12, 31716. 32551530 10.1021/acsami.0c09221

[65] R. Hayakawa , M. Petit , K. Higashiguchi , K. Matsuda , T. Chikyow , Y. Wakayama , Org. Electron. 2015, 21, 149.

[66] Y. Liu , Y. Yang , D. Shi , M. Xiao , L. Jiang , J. Tian , G. Zhang , Z. Liu , X. Zhang , D. Zhang , Adv. Mater. 2019, 31, 1902576. 10.1002/adma.201902576 31532883

[67] J. Tian , L. Fu , Z. Liu , H. Geng , Y. Sun , G. Lin , X. Zhang , G. Zhang , D. Zhang , Adv. Funct. Mater. 2019, 29, 1807176.

[68] J. Tian , Z. Liu , C. Wu , W. Jiang , L. Chen , D. Shi , X. Zhang , G. Zhang , D. Zhang , Adv. Mater. 2021, 33, 2005613. 10.1002/adma.202005613 33448055

[69] M. Carroli , A. G. Dixon , M. Herder , E. Pavlica , S. Hecht , G. Bratina , E. Orgiu , P. Samori , Adv. Mater. 2021, 33, 2007965. 10.1002/adma.202007965 33656201

[70] V. Podzorov , M. E. Gershenson , Phys. Rev. Lett. 2005, 95, 016602. 16090641 10.1103/PhysRevLett.95.016602

[71] Y. Guo , C.-A. Di , S. Ye , X. Sun , J. Zheng , Y. Wen , W. Wu , G. Yu , Y. Liu , Adv. Mater. 2009, 21, 1954.

[72] C. Feng , T. Mei , X. Hu , N. Pavel , Org. Electron. 2010, 11, 1713.

[73] W. Wang , D. Ma , Q. Gao , IEEE Trans Electron Devices 2012, 59, 1510.

[74] M. Yi , M. Xie , Y. Shao , W. Li , H. Ling , L. Xie , T. Yang , Q. Fan , J. Zhu , W. Huang , J. Mater. Chem. C 2015, 3, 5220.

[75] X. Ren , P. K. L. Chan , Appl. Phys. Lett. 2014, 104, 113302.

[76] H. Wang , Z. Ji , L. Shang , Y. Chen , M. Han , X. Liu , Y. Peng , M. Liu , Org. Electron. 2011, 12, 1236.

[77] X. Gao , C.-H. Liu , X.-J. She , Q.-L. Li , J. Liu , S.-D. Wang , Org. Electron. 2014, 15, 2486.

[78] S. Jang , E. Hwang , Y. Lee , S. Lee , J. H. Cho , Nano Lett. 2015, 15, 2542. 25811444 10.1021/acs.nanolett.5b00105

[79] T. Han , L. Liu , M. Wei , C. Wang , X. Wu , Z. Xie , Y. Ma , Phys. Chem. Chem. Phys. 2017, 19, 17653. 28671198 10.1039/c7cp02589k

[80] M. Kim , H. Seong , S. Lee , H. Kwon , S. G. Im , H. Moon , S. Yoo , Sci. Rep. 2016, 6, 30536. 27457189 10.1038/srep30536 PMC4960596

[81] S. Hong , J. Park , J. J. Lee , S. Lee , K. Yun , H. Yoo , S. Kim , NPG Asia Mater. 2021, 13, 18.

[82] S.-T. Han , Y. Zhou , L. Zhou , Y. Yan , L.-B. Huang , W. Wu , V. A. L. Roy , J. Mater. Chem. C 2015, 3, 3173.

[83] W. L. Leong , N. Mathews , S. Mhaisalkar , Y. M. Lam , T. Chen , P. S. Lee , J. Mater. Chem. 2009, 19, 7354.

[84] L. Zhou , S. T. Han , S. Shu , J. Zhuang , Y. Yan , Q. J. Sun , Y. Zhou , V. A. L. Roy , ACS Appl. Mater. Interfaces 2017, 9, 34101. 28891295 10.1021/acsami.7b07486

[85] Y. J. Jeong , D. J. Yun , S. H. Kim , J. Jang , C. E. Park , ACS Appl. Mater. Interfaces 2017, 9, 11759. 28287701 10.1021/acsami.7b02365

[86] L. X. Zhang , X. Gao , J. J. Lv , Y. N. Zhong , C. Xu , J. L. Xu , S. D. Wang , ACS Appl. Mater. Interfaces 2019, 11, 40366. 31595743 10.1021/acsami.9b15342

[87] F. Shiono , H. Abe , T. Nagase , T. Kobayashi , H. Naito , Org. Electron. 2019, 67, 109.

[88] T. T. Dao , H. Sakai , K. Ohkubo , S. Fukuzumi , H. Murata , Org. Electron. 2020, 77, 105505.

[89] T.-Y. Huang , C.-H. Chen , C.-C. Lin , Y.-J. Lee , C.-L. Liu , G.-S. Liou , J. Mater. Chem. C 2019, 7, 11014.

[90] Q. Li , T. Li , Y. Zhang , Y. Yu , Z. Chen , L. Jin , Y. Li , Y. Yang , H. Zhao , J. Li , J. Yao , Org. Electron. 2020, 77, 105461.

[91] W. Lin , G. Chen , E. Li , H. Xie , G. Peng , W. Yu , H. Chen , T. Guo , Org. Electron. 2020, 86, 105869.

[92] Y. J. Jeong , D. J. Yun , S. H. Noh , C. E. Park , J. Jang , ACS Nano 2018, 12, 7701. 30024727 10.1021/acsnano.8b01413

[93] M.-Y. Liao , M. H. Elsayed , C.-L. Chang , Y.-C. Chiang , W.-Y. Lee , W.-C. Chen , H.-H. Chou , C.-C. Chueh , ACS Appl. Nano Mater. 2021, 3, 1708.

[94] J. Y. Chen , Y. C. Chiu , Y. T. Li , C. C. Chueh , W. C. Chen , Adv. Mater. 2017, 29, 1702217. 10.1002/adma.201702217 28640532

[95] E. Ercan , J. Y. Chen , C. C. Shih , C. C. Chueh , W. C. Chen , Nanoscale 2018, 10, 18869. 30277243 10.1039/c8nr06396f

[96] Y. H. Chang , C. W. Ku , Y. H. Zhang , H. C. Wang , J. Y. Chen , Adv. Funct. Mater. 2020, 30, 2000764.

[97] M. Y. Liao , Y. C. Chiang , C. H. Chen , W. C. Chen , C. C. Chueh , ACS Appl. Mater. Interfaces 2020, 12, 36398. 32700518 10.1021/acsami.0c10587

[98] W. C. Yang , Y. C. Chiang , J. Y. Lam , T. H. Chuang , E. Ercan , C. C. Chueh , W. C. Chen , Adv. Electron. Mater. 2020, 6, 2000458.

[99] W. C. Yang , Y. C. Lin , M. Y. Liao , L. C. Hsu , J. Y. Lam , T. H. Chuang , G. S. Li , Y. F. Yang , C. C. Chueh , W. C. Chen , ACS Appl. Mater. Interfaces 2021, 13, 20417. 33886254 10.1021/acsami.1c03402

[100] A. Star , Y. Lu , K. Bradley , G. Gruner , Nano Lett. 2004, 9, 1587.

[101] S.-W. Cheng , T. Han , T.-Y. Huang , Y.-H. C. Chien , C.-L. Liu , B. Z. Tang , G.-S. Liou , ACS Appl. Mater. Interfaces 2018, 10, 18281. 29733198 10.1021/acsami.8b02560

[102] S. W. Cheng , T. Han , T. Y. Huang , Y. H. Chang Chien , C. L. Liu , B. Z. Tang , G. S. Liou , ACS Appl. Mater. Interfaces 2018, 10, 18281. 29733198 10.1021/acsami.8b02560

[103] C. H. Chen , Y. Wang , H. Tatsumi , T. Michinobu , S. W. Chang , Y. C. Chiu , G. S. Liou , Adv. Funct. Mater. 2019, 29, 1902991.

[104] C. Y. Ke , M. N. Chen , M. H. Chen , Y. T. Li , Y. C. Chiu , G. S. Liou , Adv. Funct. Mater. 2021, 31, 2101288.

[105] H. Ling , J. Lin , M. Yi , B. Liu , W. Li , Z. Lin , L. Xie , Y. Bao , F. Guo , W. Huang , ACS Appl. Mater. Interfaces 2016, 8, 18969. 27363281 10.1021/acsami.6b03792

[106] M.-N. Chen , S.-W. Chang , S. P. Prakoso , Y.-T. Li , K.-L. Chen , Y.-C. Chiu , ACS Appl. Mater. Interfaces 2021, 13, 44656. 34506100 10.1021/acsami.1c12742

[107] C. C. Shih , Y. C. Chiang , H. C. Hsieh , Y. C. Lin , W. C. Chen , ACS Appl. Mater. Interfaces 2019, 11, 42429. 31625392 10.1021/acsami.9b14628

[108] T. Xu , S. Guo , W. Qi , S. Li , M. Xu , W. Wang , ACS Appl. Mater. Interfaces 2020, 12, 21952. 32319288 10.1021/acsami.0c01162

[109] Y.-C. Chen , Y.-C. Lin , H.-C. Hsieh , L.-C. Hsu , W.-C. Yang , T. Isono , T. Satoh , W.-C. Chen , J. Mater. Chem. C 2021, 9, 1259.

[110] E. Ercan , Y. C. Lin , C. K. Chen , Y. K. Fang , W. C. Yang , Y. F. Yang , W. C. Chen , J. Polym. Sci. 2021, 13.

[111] C.-F. Lin , Y.-C. Lin , W.-C. Yang , L.-C. Hsu , E. Ercan , C.-C. Hung , Y.-Y. Yu , W.-C. Chen , Adv. Electron. Mater. 2021, 8, 2100655.

[112] Y. F. Yang , Y. C. Chiang , Y. C. Lin , G. S. Li , C. C. Hung , W. C. Chen , Adv. Funct. Mater. 2021, 31, 2012174.

[113] L. Zhang , T. Wu , Y. Guo , Y. Zhao , X. Sun , Y. Wen , G. Yu , Y. Liu , Sci. Rep. 2013, 3, 1080. 23326636 10.1038/srep01080 PMC3546321

[114] Y. C. Chiang , C. C. Hung , Y. C. Lin , Y. C. Chiu , T. Isono , T. Satoh , W. C. Chen , Adv. Mater. 2020, 32, 2002638. 10.1002/adma.202002638 32700349

[115] C. C. Hung , Y. C. Chiang , Y. C. Lin , Y. C. Chiu , W. C. Chen , Adv. Sci 2021, 8, 2100742. 10.1002/advs.202100742 PMC8373107 34096194

[116] Y.-S. Lin , Y.-C. Lin , W.-C. Yang , G.-S. Li , E. Ercan , C.-C. Hung , W.-C. Chien , W.-C. Chen , Adv. Electron. Mater. 2021, 8, 2100798.

[117] J. Zhuang , W. S. Lo , L. Zhou , Q. J. Sun , C. F. Chan , Y. Zhou , S. T. Han , Y. Yan , W. T. Wong , K. L. Wong , V. A. Roy , Sci. Rep. 2015, 5, 14998. 26449199 10.1038/srep14998 PMC4598868

[118] C. Sun , Z. Lin , W. Xu , L. Xie , H. Ling , M. Chen , J. Wang , Y. Wei , M. Yi , W. Huang , J. Phys. Chem. C 2015, 119, 18014.

[119] L. Du , X. Luo , W. Lv , F. Zhao , Y. Peng , Y. Tang , Y. Wang , Phys. Chem. Chem. Phys. 2016, 18, 13108. 27113427 10.1039/c6cp00432f

[120] C. Qian , J. Sun , L.-A. Kong , Y. Fu , Y. Chen , J. Wang , S. Wang , H. Xie , H. Huang , J. Yang , Y. Gao , ACS Photonics 2017, 4, 2573.

[121] S. Dai , Y. Zhao , Y. Wang , J. Zhang , L. Fang , S. Jin , Y. Shao , J. Huang , Adv. Funct. Mater. 2019, 29, 1903700.

[122] J. Zhang , S. Dai , Y. Zhao , J. Zhang , J. Huang , Adv. Intell. Syst. 2020, 2, 1900136.

[123] S. Song , J. Kim , S. M. Kwon , J. W. Jo , S. K. Park , Y. H. Kim , Adv. Intell. Syst. 2020, 3, 2000119.

[124] Y. Wang , Z. Lv , J. Chen , Z. Wang , Y. Zhou , L. Zhou , X. Chen , S. T. Han , Adv. Mater. 2018, 30, 1802883. 10.1002/adma.201802883 30063261

[125] S. Ham , S. Choi , H. Cho , S.-I. Na , G. Wang , Adv. Funct. Mater. 2019, 29, 1806646.

[126] D. Hao , J. Zhang , S. Dai , J. Zhang , J. Huang , ACS Appl. Mater. Interfaces 2020, 12, 39487. 32805934 10.1021/acsami.0c10851

[127] J. Y. Chen , D. L. Yang , F. C. Jhuang , Y. H. Fang , J. S. Benas , F. C. Liang , C. C. Kuo , Adv. Funct. Mater. 2021, 31, 2105911.

[128] E. Ercan , Y.-C. Lin , W.-C. Yang , W.-C. Chen , Adv. Funct. Mater. 2021, 31, 2107925.

[129] Z. Lv , M. Chen , F. Qian , V. A. L. Roy , W. Ye , D. She , Y. Wang , Z. X. Xu , Y. Zhou , S. T. Han , Adv. Funct. Mater. 2019, 29, 1902374.

[130] B. Yang , Y. Lu , D. Jiang , Z. Li , Y. Zeng , S. Zhang , Y. Ye , Z. Liu , Q. Ou , Y. Wang , S. Dai , Y. Yi , J. Huang , Adv. Mater. 2020, 32, 2001227. 10.1002/adma.202001227 32500583

[131] X. Wang , Y. Lu , J. Zhang , S. Zhang , T. Chen , Q. Ou , J. Huang , Small 2021, 17, 2005491. 10.1002/smll.202005491 33325607

[132] M. K. Kim , J. S. Lee , Adv. Mater. 2020, 32, 1907826.

[133] C. Wang , X. Zhang , W. Hu , Chem. Soc. Rev., 2020, 49, 653. 31829375 10.1039/c9cs00431a

[134] H. Xu , J. Li , B. H. K. Leung , C. C. Y. Poon , B. S. Ong , Y. Zhang , N. Zhao , Nanoscale, 2013, 5, 11850. 24126789 10.1039/c3nr03989g

[135] N. Li , Z. Lan , L. Cai , F. Zhu , J. Mater. Chem. C 2019, 7, 3711.

[136] Z. He , J. Han , X. Du , L. Cao , J. Wang , C. Zheng , H. Lin , S. Tao , Adv. Funct. Mater., 2021, 31, 2103988.

[137] J. Park , C. Lee , T. Kim , H. Kim , Y. Kim , Adv. Electron. Mater. 2021, 7, 2000932.

[138] S. Lee , C. Lee , H. Kim , Y. Kim , J. Mater. Chem. C 2020, 8, 15778.

[139] C. T. Herrera , M. J. Hong , J. G. Labram , ACS Appl. Electron. Mater. 2020, 2, 2257.

[140] Y. Chu , X. Wu , J. Lu , D. Liu , J. Du , G. Zhang , J. Huang , Adv. Sci. 2016, 3, 1500435. 10.1002/advs.201500435 PMC5069582 27812481

[141] C. Lee , H. Kim , Y. Kim , ACS Appl. Mater. Interfaces 2021, 13, 19064. 33851816 10.1021/acsami.1c00472

[142] D.-H. Lim , M. Kang , S.-Y. Jang , K. Hwang , I.-B. Kim , E. Jung , Y.-R. Jo , Y.-J. Kim , J. Kim , H. Choi , T.-W. Kim , S. Mathur , B.-J. Kim , D.-Y. Kim , ACS Appl. Mater. Interfaces 2020, 12, 25066. 32297509 10.1021/acsami.0c02229

[143] Y. Chen , Y. Chu , X. Wu , W. Ou-Yang , J. Huang , Adv. Mater. 2017, 29, 1704062. 10.1002/adma.201704062 29027731

[144] D. Li , J. Du , Y. Tang , K. Liang , Y. Wang , H. Ren , R. Wang , L. Meng , B. Zhu , Y. Li , Adv. Funct. Mater. 2021, 31, 2105887.

[145] B. Yang , Y. Wang , L. Li , J. Zhang , J. Wang , H. Jiao , D. Hao , P. Guo , S. Zeng , Z. Hua , J. Huang . Adv. Funct. Mater. 2021, 31, 2103787.

[146] C. Wang , D. T. Duong , K. Vandewal , J. Rivnay , A. Salleo , Phys. Rev. B Condens. Matter 2015, 91, 085205.

[147] A. Y. B. Meneau , Y. Olivier , T. Backlund , M. James , D. W. Breiby , J. W. Andreasen , H. Sirringhaus , Adv. Funct. Mater. 2016, 26, 2326.

[148] A. R. Chew , R. Ghosh , V. Pakhnyuk , J. Onorato , E. C. Davidson , R. A. Segalman , C. K. Luscombe , F. C. Spano , A. Salleo , Adv. Funct. Mater. 2018, 28, 1804142.

[149] R. Ghosh , C. K. Luscombe , M. Hambsch , S. C. B. Mannsfeld , A. Salleo , F. C. Spano , Chem. Mater. 2019, 31, 7033.

